# Efficient purging of deleterious mutations contributes to the survival of a rare conifer

**DOI:** 10.1093/hr/uhae108

**Published:** 2024-04-11

**Authors:** Yi Wang, Yongzhi Yang, Zhitong Han, Jialiang Li, Jian Luo, Heng Yang, Jingge Kuang, Dayu Wu, Shiyang Wang, Sonam Tso, Tsam Ju, Jianquan Liu, Susanne S Renner, Mao Kangshan

**Affiliations:** Key Laboratory of Bio-Resource and Eco-Environment of Ministry of Education, Sichuan Zoige Alpine Wetland Ecosystem National Observation and Research Station, College of Life Sciences, State Key Laboratory of Hydraulics and Mountain River Engineering, Sichuan University, Chengdu 610065, China; State Key Laboratory of Herbage Improvement and Grassland Agro-ecosystems, College of Ecology, Lanzhou University, Lanzhou 730000, China; Key Laboratory of Bio-Resource and Eco-Environment of Ministry of Education, Sichuan Zoige Alpine Wetland Ecosystem National Observation and Research Station, College of Life Sciences, State Key Laboratory of Hydraulics and Mountain River Engineering, Sichuan University, Chengdu 610065, China; Key Laboratory of Bio-Resource and Eco-Environment of Ministry of Education, Sichuan Zoige Alpine Wetland Ecosystem National Observation and Research Station, College of Life Sciences, State Key Laboratory of Hydraulics and Mountain River Engineering, Sichuan University, Chengdu 610065, China; Xizang Key Laboratory of Forest Ecology in Plateau Area of Ministry of Education, National Key Station of Field Scientific Observation & Experiment of Alpine Forest Ecology System in Nyingchi, Research Institute of Xizang Plateau Ecology, Xizang Agriculture & Animal Husbandry University, Nyingchi 860000, China; Key Laboratory of Bio-Resource and Eco-Environment of Ministry of Education, Sichuan Zoige Alpine Wetland Ecosystem National Observation and Research Station, College of Life Sciences, State Key Laboratory of Hydraulics and Mountain River Engineering, Sichuan University, Chengdu 610065, China; Key Laboratory of Bio-Resource and Eco-Environment of Ministry of Education, Sichuan Zoige Alpine Wetland Ecosystem National Observation and Research Station, College of Life Sciences, State Key Laboratory of Hydraulics and Mountain River Engineering, Sichuan University, Chengdu 610065, China; Key Laboratory of Bio-Resource and Eco-Environment of Ministry of Education, Sichuan Zoige Alpine Wetland Ecosystem National Observation and Research Station, College of Life Sciences, State Key Laboratory of Hydraulics and Mountain River Engineering, Sichuan University, Chengdu 610065, China; Key Laboratory of Bio-Resource and Eco-Environment of Ministry of Education, Sichuan Zoige Alpine Wetland Ecosystem National Observation and Research Station, College of Life Sciences, State Key Laboratory of Hydraulics and Mountain River Engineering, Sichuan University, Chengdu 610065, China; School of Ecology and Environment, Tibet University, Lhasa 850000, China; School of Ecology and Environment, Tibet University, Lhasa 850000, China; Key Laboratory of Bio-Resource and Eco-Environment of Ministry of Education, Sichuan Zoige Alpine Wetland Ecosystem National Observation and Research Station, College of Life Sciences, State Key Laboratory of Hydraulics and Mountain River Engineering, Sichuan University, Chengdu 610065, China; Department of Biology, Washington University, Saint Louis, MO 63130, USA; Key Laboratory of Bio-Resource and Eco-Environment of Ministry of Education, Sichuan Zoige Alpine Wetland Ecosystem National Observation and Research Station, College of Life Sciences, State Key Laboratory of Hydraulics and Mountain River Engineering, Sichuan University, Chengdu 610065, China; School of Ecology and Environment, Tibet University, Lhasa 850000, China

## Abstract

Cupressaceae is a conifer family rich in plants of horticultural importance, including *Cupressus*, *Chamaecyparis*, *Juniperus*, and *Thuja*, yet genomic surveys are lacking for this family. *Cupressus gigantea*, one of the many rare conifers that are threatened by climate change and anthropogenic habitat fragmentation, plays an ever-increasing role in ecotourism in Tibet. To infer how past climate change has shaped the population evolution of this species, we generated a *de novo* chromosome-scale genome (10.92 Gb) and compared the species’ population history and genetic load with that of a widespread close relative, *C. duclouxiana*. Our demographic analyses, based on 83 re-sequenced individuals from multiple populations of the two species, revealed a sharp decline of population sizes during the first part of the Quaternary. However, populations of *C. duclouxiana* then started to recover, while *C. gigantea* populations continued to decrease until recently. The total genomic diversity of *C. gigantea* is smaller than that of *C. duclouxiana*, but contrary to expectations, *C. gigantea* has fewer highly and mildly deleterious mutations than *C. duclouxiana*, and simulations and statistical tests support purifying selection during prolonged inbreeding as the explanation. Our results highlight the evolutionary consequences of decreased population size on the genetic burden of a long-lived endangered conifer with large genome size and suggest that genetic purging deserves more attention in conservation management.

## Introduction

**Figure 1:**
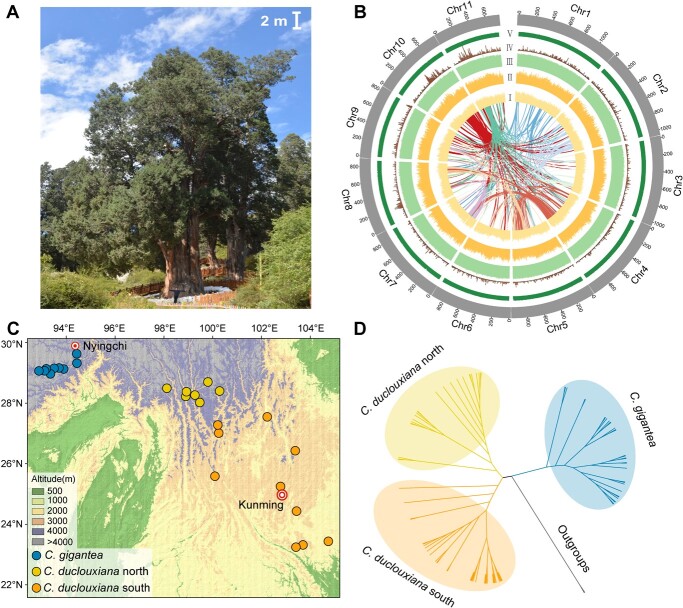
Habit, genomic landscape, geographic sampling, and phylogeny for *Cupressus gigantea*. **A** The so-called King Cypress, one of the largest known individuals at Nyingchi. **B** Genomic landscape of the 11 assembled chromosomes. Track V, GC content; track IV, gene density; track III, distribution of repeat elements; track II, distribution of Ty3-Gypsy elements; track I, distribution of Ty1-Copia elements; center, intra-genome collinear blocks connected by curved lines. **C** Sample locations of the 9 sampled *C. gigantea* populations and the 17 *C. duclouxiana* populations. **D** A neighbor-joining phylogenetic tree of all sampled individuals based on identity-by-state (IBS) genetic distances.

Many conifers are important as sources of timber, in landscaping, and in the cultures of people around the world. Some, such as species of *Cupressus*, *Chamaecyparis*, *Juniperus*, and *Thuja*, have been the subject of selection for ornamental purposes, leading to the development of hundreds of cultivars [1]. Others, such as the common cypress, *Cupressus sempervirens*, are highly praised trees with a rich historical significance in cultures across West Asia, Asia Minor, the Mediterranean basin, and North Africa [2]. In Tibet, species of *Cupressus* have been used for temple construction since the Bronze Age, and there is evidence that Cupressaceae forests transitioned into desert pastures at some point within the last 5000 years [3]. Among the culturally most important species is *Cupressus gigantea* W. C. Cheng & L. K. Fu, locally known as the Tsangpo River cypress, which has a narrow distribution in the dry valleys of the Yarlung Tsangpo and Nyang rivers in the southern Qinghai–Tibet Plateau (Fig. 1). This endemic cypress is classified as ‘Vulnerable’ in the IUCN Red List [4] and a ‘First-class national key protected wild plant’ in Chinese rare species lists [5]. It is the highest and largest tree living 3000 m above sea level: Mature individuals reach between 30 and 45 m in height, with diameters of 3–6 m [6]. In the valleys where it occurs, *C. gigantea* and *Pinus densata*, another conifer with smaller size, are the only two species of trees that can provide the timber for diverse artificial construction [3, 7]. In addition, the branchlets of *C. gigantea* are one of the raw materials for the production of special incense, which is used by the Tibetans in their daily lives and religious practices [8].

Here we focus on the genetics of *C. gigantea*, specifically on its demographic history, genetic diversity, and genetic load (defined as the reduction of population fitness due to the fixation of deleterious mutations [9]). As population sizes decrease, inbreeding increases, with negative effects on genetic diversity, making populations more vulnerable to external threats [10, 11]. The expected negative feedback loop continues by increasing the probability of stochastic demographic events and genetic drift [12]. Population genetic theory predicts that, in small populations, recessive deleterious mutations tend to accumulate and increase the risk of extinction [9, 13]. On the other hand, continuous inbreeding results in the increased expression of (partially) recessive deleterious mutations, which creates the potential for purifying selection to remove these mutations. This process, known as genetic purging, depends on the degree of dominance and the magnitude of the deleterious effects [14]. For plants, more recent studies have examined the genetic effects after prolonged population decline in a rare Asian Betulaceae, *Ostrya rehderiana*, and its widespread close relative, *O. chinensis* [15], in the Chinese Tertiary relict species *Dipteronia dyeriana* and *D. sinensis* [16], and in Chinese endemic apricots (*Prunus hongpingensis* and *P. zhengheensis*) [17]. No study so far has focused on the genomic effects of population bottlenecks in conifers, likely because of their huge genomes.

Here, we sequenced and assembled a high-quality genome for *C. gigantea*, which has a large genome size of around 11 Gb, and then re-sequenced 31 additional *C. gigantea* and 52 *Cupressus duclouxiana* individuals across their distributional ranges (Fig. 1) to identify genome-wide genetic variations. *Cupressus duclouxiana* diverged from *C. gigantea* about 8 million years ago (Mya) [18] and is widespread between 1400 and 3300 m in Yunnan and southwestern Sichuan (Fig. 1). Based on these genomic data, we aimed to address the following questions: (i) Did the demography of two species respond similarly to historical climatic oscillations or more recent disturbance by humans? If not, why might their demographic histories differ? (ii) What is the pattern of accumulation of deleterious mutations and genetic purging in the common versus the rare species?

## Results

### Genome evolution of *Cupressus gigantea*

Based on *k*-mer frequency analysis with ~1380 Gb (~113.04× depth) DNBSEQ short reads, the genome size of *C. gigantea* was estimated to be 10.38 Gb (Table 1; Supplementary Data Fig. S1 and Supplementary Data Table S1). To obtain a high-quality of genome for *C. gigantea*, we first generated ~1212 Gb (~117× depth) Nanopore long sequencing reads, resulting in a primary genome of 10.92 Gb. This assembly contained 18 562 contigs with contig N50 of 1.61 Mb (Table 1; Supplementary Data Table S2). We then used ~1152 Gb Hi-C reads (~111× depth) to assist the assembly correction. Consequently, nearly 94% (10.26 Gb) of the assembled contigs were anchored to 11 chromosomes. The super-scaffold N50 was improved to 917.08 Mb, and the longest chromosome contains 1189.33 million bases (Table 1, Fig. 1B; Supplementary Data Fig. S2). Based on BUSCO estimation, 1296 of 1614 core genes were complete (Supplementary Data Table S3). In addition, ~99.87% of short reads and 90.02% of RNA-seq reads could be mapped onto the assembly. Together these results indicate the relatively high completeness and continuity of the *C. gigantea* genome (Supplementary Data Tables S2 and S4).

**Table 1:** Statistics for genome sequencing of *Cupressus gigantea*.

**Category**	**Item**	**Statistic**
**Sequencing**	DNBseq data (Gb)/depth (×)	1380.98/113.04
	Nanopore data (Gb)/depth (×)	1212.20/116.78
	Hi-C data (Gb)/depth (×)	1152.33/111.01
**Assembly features**	Estimated genome size (Gb)	10.38
	Assembly genome size (Gb)	10.92
	Number of contigs	18 562
	Contig N50 (Mb)	1.61
	Number of scaffolds	605
	Scaffold N50 (Mb)	917.08
	Longest scaffold (Mb)	1189.33
	Chromosome-scale scaffolds (Gb)	10.26 (94.96%)
	GC content (%)	34.90
**Annotation**	Predicted gene number	35 384
	Functional gene number	31 306
	Repetitive elements content (%)	88.62

By combining *ab initio*, homology, and transcriptome prediction strategies, a total of 35 384 hypothetical protein-coding genes were annotated. Repetitive sequences make up a large portion (~9.68 Gb) of the *C. gigantea* genome, with the most abundant type being long terminal repeat retrotransposons (LTR-RTs) (Table 1; Supplementary Data Tables S5–S7). The expansion of LTR-RTs occurred rapidly between 1 and 2 Mya, a timeframe notably younger than previously estimated gymnosperm genomes [19], pointing to a relatively unique TE expansion in *C. gigantea* (Supplementary Data Fig. S3). The distribution of synonymous substitution rate (*K*_s_) and the distance-transversion rate at 4-fold degenerate sites (4Dtv) indicate that the *C. gigantea* genome shares the seed plant whole-genome duplication (WGD) [20], but no additional duplication (Supplementary Data Fig. S4). A total of 2558 expanded gene families and 86 significantly expanded families were present in *C. gigantea* relative to *Sequoiadendron giganteum*. We also identified 694 gene families unique to *C. gigantea*. Functional enrichment analysis indicates that these expanded and unique gene families are mainly associated with flavone and flavanol biosynthesis, hypoxia, and cold stress response (Supplementary Data Fig. S5; Supplementary Data Tables S8–S11).

### Population structure and demographic history

Overall, 83 individuals (32 individuals from 9 populations of *C. gigantea* and 51 individuals from 17 populations of *C. duclouxiana*) were sampled and used for population genetic analyses (Fig. 1C; Supplementary Data Table S12). We generated 14.78 Tb data, resulting in an average sequencing depth of ~15× for each accession (Supplementary Data Table S13). Based on the mapping results, we obtained ~1390 million high-quality SNPs, ~97.19% of them located in intergenic regions (Supplementary Data Fig. S6).

Based on linkage disequilibrium (LD)-pruned SNPs, we first clustered individuals using phylogenetic reconstruction analysis. The neighbor-joining (NJ) tree supported the deep split between two species, and *C. duclouxiana* was then further divided into a northern and a southern lineage (Fig. 1D). Clustering by principal component analysis (PCA) also supported three distinct groups (Supplementary Data Fig. S7). Genome-wide LD varies markedly among the species, with *C. gigantea* having a slower LD decay, with half the maximum *r*^2^ not attained until ~350 kb, whereas in *C. duclouxiana* half the maximum *r*^2^ was attained at ~185 kb (Supplementary Data Fig. S8).

**Figure 2:**
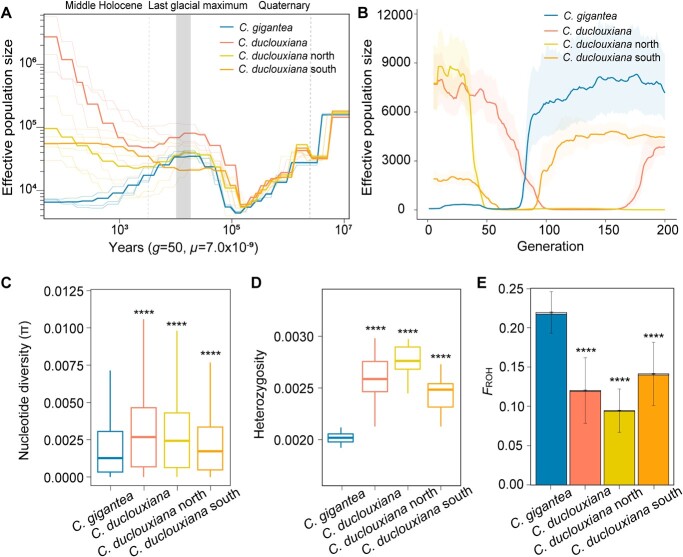
Demographic history, genetic diversity, and estimates of inbreeding. **A** The demographic history was inferred using SMC++. The time scale on the *x* axis was calculated based on a mutation rate per generation (*μ*) of 7.0 × 10^−9^ and a generation time (*g*) of 50 years. The pale extra lines represent randomized replicates. The last glacial maximum is indicated by gray vertical bars. The grey dotted lines depict the onset of the Quaternary and of the middle Holocene. **B** The demographic history was inferred using GONE. The light background colors correspond to the upper and lower bounds of the 95% confidence intervals. **C*–*E** Boxplots showing (**C**) genetic diversity (π), (**D**) whole-genome heterozygosity for each individual, and (**E**) inbreeding estimated from the genome proportion with runs of homozygosity (*F*_ROH_). Colored bars depict the total proportion of the genome with ROH longer than 100 kb and the open bars show ROH longer than 1 Mb. *P* values for comparisons were obtained from Welch’s *t*-tests, with asterisks denoting the significance level (^****^P < 0.0001). Comparisons were conducted between *Cupressus gigantea* and *C. duclouxiana*, the latter either as a single entity or instead separated into its northern and southern populations.

Based on whole-genome data, we further explored the demographic history of *C. gigantea* and *C. duclouxiana*. Results from SMC++ analysis of changes in effective population size (*N*_e_) over the past 10 million years (Fig. 2A) show that both species endured similar declines during the early Quaternary and then started to re-expand until the beginning of the Holocene (11 700 years ago), when the *N*_e_ of *C. gigantea* began to decline again, never to recover until the present [21]. This inference was also supported by Stairway Plot analyses (Supplementary Data Fig. S9). A GONE analysis of the species’ more recent population history indicated that, in contrast to the population recovery of *C. duclouxiana*, the *N*_e_ of *C. gigantea* has continued to decrease for the past ~6000 years. This period spans ~120 generations, assuming a generation time of 50 years (Fig. 2B).

### Genetic diversity and inbreeding in *Cupressus gigantea*

We next tested how the reduced population sizes have influenced the two species’ genetic diversity and heterozygosity. *Cupressus gigantea* has significantly lower genetic diversity (π = 0.00201) and heterozygosity (0.00201, individual-based) than *C. duclouxiana* (π = 0.00308, *P* < 0.0001; heterozygosity = 0.00257, *P* < 0.0001; Fig. 2C and D; *t*-test). In addition, *F*_ROH_ [the fraction of the genome in runs of homozygosity (ROH)], a genomic measure of inbreeding (ROH length >100 kb), differed markedly between the species. On average, ROH regions constituted 21.93% of the *C. gigantea* genome but only 12.02% of the *C. duclouxiana* genome (Fig. 2E; Supplementary Data Figs S10 and S11), indicating a higher level of inbreeding in *C. gigantea*. Using a threshold for ROH length of >1 Mb to evaluate recent inbreeding levels [15, 22], we found that 0.2198% of the *C. gigantea* and 0.1171% of the *C. duclouxiana* genome consisted of such long ROH regions (Fig. 2E). Individuals’ whole-genome heterozygosity was also negatively correlated with *F*_ROH_ in both *C. gigantea* (*r*^2^ = 37.34%, *P* < 0.00012) and *C. duclouxiana* (*r*^2^ = 76.88%, *P* < 2.2e−16) (Supplementary Data Fig. S12).

### *Cupressus gigantea* has fewer deleterious mutations than the widespread *C. duclouxiana*, likely due to increasing inbreeding and purifying selection

**Figure 3:**
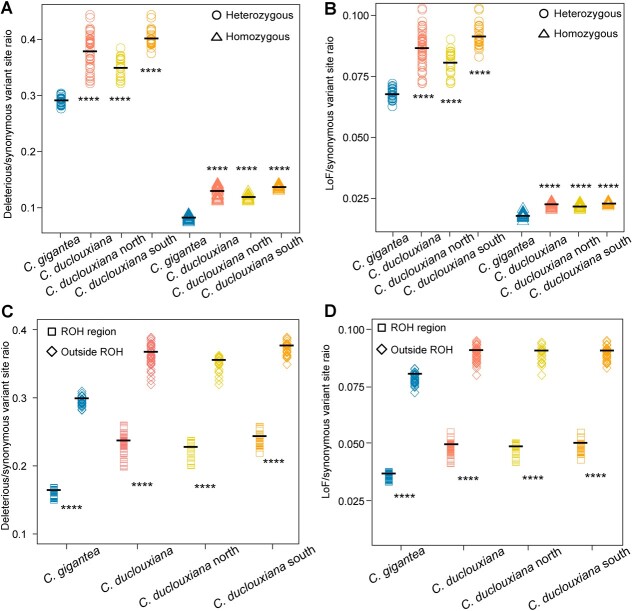
Characterization of the genetic load of *Cupressus gigantea* and *C. duclouxiana*. **A**, **B** Ratio of derived deleterious (**A**) and LoF (**B**) variants to derived synonymous variants in heterozygous (circles) and homozygous (triangles) tracts per individual. Horizontal bars represent the average values. *P* values for comparisons were obtained from Welch’s *t*-tests, with asterisks denoting the significance level (^****^P < 0.0001). Comparisons were conducted between *C. gigantea* and *C. duclouxiana*, the latter either as a single entity or instead separated into its northern and southern populations. **C**, **D** Ratio of derived deleterious (**C**) and LoF (**D**) variants to derived synonymous variants inside ROH regions (squares) and outside ROH regions (rhombi) per individual. Horizontal bars represent the average values. *P* values for comparisons were obtained from Welch’s *t*-tests, with asterisks denoting the significance level (^****^P < 0.0001; a comparison was conducted between in-ROH regions and outside ROH).

To estimate the genetic load of *C. gigantea* and *C. duclouxiana*, we first calculated the π (0-fold degenerate variants)/π (4-fold degenerate variants) ratio. We found a lower ratio in *C. gigantea* than in *C. duclouxiana* (Supplementary Data Fig. S13), suggesting that *C. gigantea* is under stronger purifying selection. To further test this, we assessed the genetic load by analyzing the accumulation of deleterious derived alleles. For this, SNPs in coding sequences were categorized into four groups based on their impact on gene function: synonymous, tolerated, deleterious, and loss of function (LoF). In both species, most deleterious derived alleles were maintained in a heterozygous state, and there were fewer such alleles in *C. gigantea* than *C. duclouxiana* (Supplementary Data Fig. S14). Since the mutation rate of different species may be different, we used the number of derived synonymous mutations for normalization by comparing the ratio of derived functional variants (including LoF, deleterious and tolerated variants) to derived synonymous mutations at heterozygous sites and homozygous sites and found reduced LoF and missense variants in *C. gigantea* compared with *C. duclouxiana* (Fig. 3A and B; Supplementary Data Fig. S15). Moreover, the ROHs had fewer LoF and deleterious alleles in the two species, and *C. gigantea* carried many fewer LoF and deleterious alleles in ROH regions than did *C. duclouxiana* (Fig. 3C and D).

**Figure 4:**
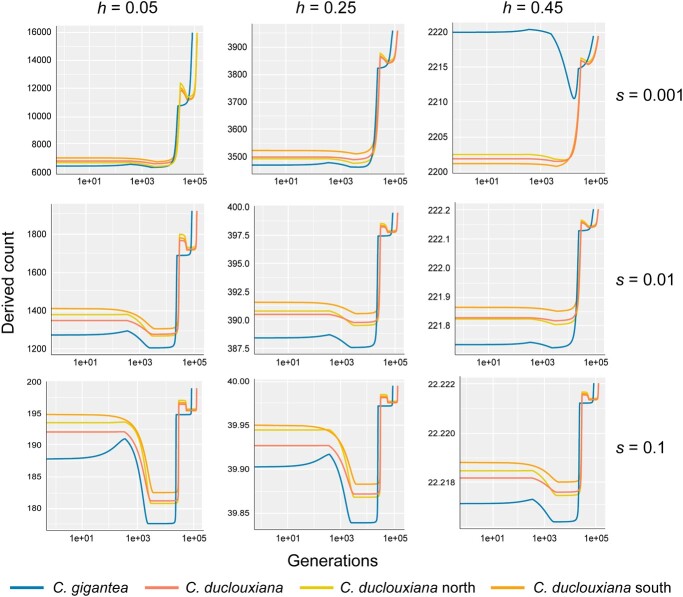
Predicted evolution of the deleterious burden for *Cupressus gigantea* and *C. duclouxiana*. The *x* axis corresponds to the generations before the present as a decimal logarithm. Panels depict different combinations the dominance coefficient (*h*) and the homozygous deleterious effect (*s*) based on the population demographic history, always assuming a haploid mutation rate of λ = 1. *Cupressus duclouxiana* was treated either as a single entity or instead separated into its northern and southern populations.

To further test to what extent the detected purging of deleterious mutations in *C. gigantea* might be the result of prolonged inbreeding, we predicted the dynamics of deleterious derived alleles, using different values for the dominance coefficient (*h*) and the homozygous deleterious effect (*s*) (Fig. 4). When considering scenarios consistent with the population demographic history, our simulation suggested that, after the first population decline (~6–0.15 Mya; Fig. 2A), purging produced a larger reduction of deleterious mutations in *C. gigantea*, particularly for mildly (*s* = 0.01) and strongly (*s* = 0.1) recessive deleterious mutations. Conversely, for weakly deleterious (*s* = 0.001) mutations with roughly additive effect (*h* = 0.45), reductions in the *N*_e_ resulted in an increased mutation burden in the long term*.* However, within the time scale represented in these predictions, the increase in the genetic load due to weakly deleterious mutations with roughly additive effect was smaller than the reduction of purging observed for the recessive deleterious mutations (*h* < 0.25). Lastly, we predicted the purging dynamics in extremely bottlenecked populations. The results showed that when populations became extremely small, e.g. *N*_e_ *=* 1000, the accumulation of deleterious mutations soared due to drift (Supplementary Data Fig. S16).

## Discussion

Our study reveals demographic insights on two species of *Cupressus*, a genus rich in species of cultural and economic significance. By providing a chromosome-level genome (10.92 Gb, scaffold N50 = 917.08 Mb) of *C. gigantea*, a large, threatened conifer that today is restricted to the dry valleys of the Yarlung Tsangpo River and Nyang River on the Qinghai–Tibet Plateau, we add an important genetic resource for the future protection of conifer germplasm. In addition, our whole-genome resequencing-based population genetic analysis of *C. gigantea* and its widespread relative *C. duclouxiana* revealed the decreased genetic diversity of the former species. This is consistent with our estimate that at present the effective population size (*N*_e_) of *C. gigantea* is only around 0.2% that of *C. duclouxiana* (Fig. 2A)*.* Our demographic reconstruction showed that both species underwent similar population decline and recovery from the Pliocene to the Quaternary, reflecting major climatic fluctuations since the late Miocene. However, *C. gigantea* experienced sharper population reductions after the Naynayxungla glaciation (0.8–0.5 Mya) [21], resulting in a consistently smaller *N*_e_ than *C. duclouxiana*. Although the population sizes of both species recovered by ~0.15 Mya, the population size of *C. gigantea* increased more slowly than that of *C. duclouxiana*. Even at the peak of population growth (~30 000 years ago), the *N*_e_ of *C. gigantea* was only ~43.03% of that of *C. duclouxiana*. The two species’ different deep demographic histories may reflect differences in their habitats in terms of climate, altitude, and topology: *C. duclouxiana* is mainly distributed in the lower-altitude Hengduan Mountains within alternate valleys and mountains (Fig. 1C), rather than the central highland, and it may therefore have been less impacted by the Pleistocene glaciations. Climate refugia in the valleys of the Hengduan Mountains may also have helped its population expansion [23]. By contrast, *C. gigantea* may have been restricted to the higher Qinghai–Tibet Plateau, which likely suffered more severely from the Pleistocene climatic fluctuations. Even during the interglacial climate warming periods, the proximity to glaciers and the restricted availability of suitable habitats could have hampered the recovery of *C. gigantea* [21, 24].

After the Last Glacial Maximum, the populations of both species declined, but unlike *C. duclouxiana*, the population size of *C. gigantea* never recovered and kept falling through the Holocene according to SMC++ (Fig. 2A). Our reconstruction of the species’ recent demographic history using GONE [25] further suggested that *C. gigantea* experienced a sharp reduction of *N*_e_ starting ~6000 years ago (Fig. 2B), while the *N*_e_ of *C. duclouxiana* recovered. The two species’ contrasting recent demographic histories likely result from different degrees of anthropogenic disturbance. Anthropogenic disturbance in the Yarlung Tsangpo valley is documented by Bronze Age cultural remains, including agriculture [26] and temples built from cypress wood from ~4300 years ago [7]. This likely involved the felling of *C. gigantea*, because along with *Pinus densata*, it represents one of the very few timber species in this tree-deficient region. Archaeological remains and paintings in ancient temples also support that Holocene humans cut down high-altitude timber for construction [3]. *Cupressus duclouxiana*, by contrast, mainly occurs at lower altitudes in the southern Hengduan Mountains and the Yungui Plateau, which are covered by species-rich forests that probably suffered less from monospecific logging.

Our study further reveals the effects of long-term population size decline on the genetic load in these long-lived conifers. Obtaining direct fitness estimates for woody plants, e.g. from the numbers of developing seeds following pollination, is challenging in trees that occur in remote parts of Tibet and whose cones are borne at 5–40 m above the ground. Modern studies therefore rely on genomics approaches to study the effects of inbreeding and the genetic load of trees [15–17] and rare animals [9, 13, 27]. Interestingly, we found that the more endangered species *C. gigantea* has a lower genetic load than its more widespread relative, *C*. *duclouxiana*. The most plausible explanation for this is stronger genetic purging during a severe population bottleneck in the distant past (Fig. 2A), when effective population sizes of *C. gigantea* appear to have been down to perhaps just 4416–4709 individuals, followed by a pronounced population decline from ~6000 to hundreds of individuals during the mid-Holocene (Fig. 2B). This interpretation is also supported by the lower π_0_/π_4_ ratio and fewer deleterious mutations within ROHs, suggesting a reduction of both highly and mildly deleterious mutations through prolonged inbreeding in *C. gigantea*.

Previous empirical studies of genetic purging in wild populations have found that severely deleterious variants are more likely to be purged by strong purifying selection, whereas slightly deleterious mutations tend to accumulate due to relaxed purifying selection, which eventually leads to increased genetic load [15, 28–30]. We also explored the accumulation of deleterious mutations in *C. gigantea* considering genetic drift and purging under four population size scenarios, including a severe population decline to about one-sixth of the current *N*_e_ (*N*_e_ = 1000). Under the latter scenario, the accumulation of deleterious mutations soared due to drift (Supplementary Data Fig. S16), as may have occurred in *O. rehderiana*, in which only a handful of individuals may have survived an inferred bottleneck [15]. The somewhat larger *N*_e_ of *C. gigantea* could have permitted more effective purifying selection of deleterious mutations than was possible in *O. rehderiana*.

Today, *C. gigantea* is well protected in the Gongbu Nature Reverse, which was designed specifically to protect this tree species. Moreover, we found the absence of very long ROH (lengths >1 Mb) in all sampled populations of *C. gigantea*, consistent with a previous result of low inbreeding based on transcriptome data [31]. Field observations by one of us, Jian Luo, found that *C. gigantea* is fruiting normally and producing seedlings, suggesting that populations today are not suffering from strong inbreeding depression. Thus, the long-term decreasing population size of *C. gigantea* seems to have facilitated extensive purging of deleterious alleles and contributed to the populations’ adaptation and survival.

## Materials and methods

### Plant material and genome sequencing

For genome sequencing, fresh intact young scale leaves of *C. gigantea* were collected from the Forestry Bureau’s central nursery, Nyingchi, Tibet (94°14′2″ E, 29°45′9″ N). High-quality genomic DNA was firstly isolated and extracted from these fresh young scale leaves using a modified CTAB method [32]. Regarding Nanopore sequencing, we constructed 20-kb libraries using the SQK-LSK109 kit presented by Oxford Nanopore Technologies (ONT). These libraries were subsequently processed on the PromethION platform, utilizing a total of 20 cells. A single independent complementary library with 300- to 400-bp insertions was also generated and sequenced on the DNBSEQ™ platform. To achieve chromosome-level genome assembly, two Hi-C libraries prepared with the MboI restriction enzyme were created following the procedures described previously [33] and sequenced on the DNBSEQ™ platform. Additionally, we conducted RNA sequencing (RNA-seq) for five tissues that included shoots, scale leaves, stems, cones, and roots (Supplementary Data Table S4). Briefly, total RNAs were isolated and extracted using TRIzol™ reagent (Invitrogen), followed by assessment of RNA integrity using the Agilent 2100 Bioanalyzer system (Agilent Technologies). Paired-end libraries (150 bp) were then constructed using MGIEasy RNA Library Prep Set according to the manufacturer’s protocols. Finally, we conducted the sequencing of these libraries on the MGISEQ-2000 platform.

### Genome assembly

The chromosome-level assembly of *C. gigantea* comprised the following steps: initial assembly, short reads correction, Hi-C scaffolding, and manual checking of positioning and ordering. First, all raw ONT long reads were base error-corrected by Canu (ver. 2.0) [34]. The SMARTdenovo (ver. 1.0; https://github.com/ruanjue/smartdenovo) software was then used to assemble the contigs. Next, the clean reads generated from DNBSEQ were aligned back to the assembled contigs using the Burrows–Wheeler Aligner program (BWA-MEM ver. 0.7.17) [35] and sorted by SAMtools (ver. 1.9) [36]. GATK (ver. 4.2.0) UnifiedGenotyper was employed for the identification of homozygous variants with specific criteria (base quality ≥20, mapping quality ≥40, and depth ≥ 2) and to generate a refined assembly [37]. For Hi-C scaffolding, the processed Hi-C reads were aligned to the assembled contigs via Juicer (ver. 1.5.6) [38] and BWA-MEM, utilizing default settings. Subsequently, HiC-Pro (ver. 2.7.8) was employed to assess library quality by quantifying the abundance of unique valid paired-end reads [39]. Unique mapped read pairs were preserved for downstream analysis. The 3D-DNA pipeline was employed to execute clustering, ordering, and orientation procedures, leveraging normalized Hi-C interactions as the basis [40]. Finally, the scaffolds were partitioned into 1-kb bins, and ordering and orientation were adjusted manually based on the contact maps generated by HiCPlotter software (https://github.com/kcakdemir/HiCPlotter/).

To evaluate the completeness and continuity of the assembly, we mapped the RNA-seq reads to the chromosomes using HISAT2 (ver. 2.1.0) with default settings [41]. Furthermore, we employed BUSCO (ver. 5.beta.1) to search for 1614 conserved protein models from the Embryophyta odb10 database within the genome sequences, providing additional assessment of the genome assembly quality [42].

### Genome repeat element identification and gene prediction

To annotate and analyze repetitive sequences within the *C. gigantea* genome, a dual approach combining homology-based and *de novo* methods was employed. Specifically, we utilized RepeatModeler (ver. 2.0.1) to construct a *de novo* repeat library [43]. RepeatMasker (ver. 4.1.1) [44] and RepeatProteinMask (http://www.repeatmasker.org/) were employed to create a ‘Viridiplantae’ repeat library from the Repbase database (ver. 22.12). Tandem Repeats Finder (ver. 4.09) was additionally utilized for the identification of tandem repeat elements [45].

Next, we predicted protein-coding genes within the repeat-masked *C. gigantea* genome using a combination of *ab initio*-based, homology-based, and RNA-seq-based approaches (see details in Supplementary Data). The integrated gene set was generated by EVidenceModeler (EVM; ver. 1.1.1) [46]. The functions of protein-coding genes were assigned following two strategies. Firstly, we adopted eggNOG-mapper (ver. 2) to align proteins to the eggNOG5.0 database [47]. Secondly, we performed BLASTP (E-values ≤1e−5) alignments of the predicted protein sequences against multiple databases, including Gene Ontology (GO), Kyoto Encyclopedia of Genes and Genomes (KEGG), Cluster of Orthologous Groups of proteins (COG), Non-redundant Protein Sequence Database (NR), and Swiss-Prot protein database. Results generated from these two strategies were integrated to predict the genes.

### Plant material and whole-genome resequencing of *Cupressus gigantea*, *C. duclouxiana*, and outgroups

To conduct a comparative population-genomics study, we collected leaf material from 9 wild *C. gigantea* populations (*n* = 32) and 17 wild *C. duclouxiana* populations (*n* = 51) in the southern Qinghai–Tibet Plateau (Fig. 1C; Supplementary Data Table S12). Because of the huge genomes of the species investigated in this study, resequencing encountered unprecedented challenges, including higher costs and computational demands. To detect genetic variation across the whole geographical distribution of the two species, we sampled from as many populations as possible, but only two to seven mature individuals per population [48]. In each population, the distance between sampled individuals was >100 m. Young scale leaves (~1 g per sample) were collected, rapidly desiccated using silica gel, sealed in plastic bags, and transported back to the laboratory. Additionally, we collected leaves from one *Juniperus microsperma* tree and five *Cupressus chengiana* trees as outgroup samples (Supplementary Data Table S13). Research and sample collection were both approved by the Forestry and Grassland Bureau of the Tibet Autonomous Region [as a part of the Second Tibetan Plateau Scientific Expedition and Research (STEP) program]. Permanent vouchers for this study have been deposited in the Sichuan University Museum under the accession numbers SZ02076005–SZ02076092. For each sample, genomic DNA was isolated and extracted using the Magnetic Universal Genomic DNA kit (Tiangen, China) following the provided protocols. DNA quality was evaluated using 1% agarose gels, while the concentration was determined using the Qubit^®^ DNA Assay Kit in the Qubit^®^ 3.0 Fluorometer (Invitrogen, USA). A quantity of 0.2 μg genomic DNA from each sample was used to construct a sequencing library using the NEB Next^®^ Ultra™ DNA Library Prep Kit (NEB, USA), followed by sequencing on the DNBSEQ-T7 platform. Each sample was sequenced to achieve a target depth of 15×. We used fastp (ver. 0.21.0) [49] to remove adaptors and low-quality bases and obtained clean sequencing reads with 167.96 Gb data for each sample on average for further analysis (Supplementary Data Table S13).

### Variation calling, quality control and validation

After quality control, the filtered reads of each sample were aligned to the *C. gigantea* reference genome using BWA-MEM with default parameters [35]. SAMtools was employed to convert the SAM format file into BAM format and sort the alignments based on mapping coordinates [36]. Duplicated reads, which may have been introduced during library construction, were then removed using Sambamba (ver. 0.8.3) [50]. Finally, the coverage and depth of sequence alignments were calculated using the depth program in SAMtools (Supplementary Data Table S13).

For SNP and InDel identification, we again used GATK with the HaplotypeCaller module and the GVCF mode [37]. In brief, the BAM alignment file was firstly processed through HaplotypeCaller to call haplotypes for each sample. Subsequently, a joint genotyping step was performed on genomic variant call formats (GVCFs) files using GenotypeGVCFs to consolidate variations comprehensively. The GATK-recommended hard-filtering criteria were then applied to exclude variants with low confidence (QUAL <30 || DP < 5 || QD < 2.0 || MQ < 40.0 || FS > 60.0 || SOR > 3.0 || MQRankSum < −12.5 || ReadPosRankSum < −8.0). This yielded a total of ~1390 million high-quality SNPs that served as the basis for all analyses.

### Population structure analysis

For all individuals, we further filtered out SNPs with a minor allele frequency (MAF) ≤0.05 and missing rate ≥10%. To mitigate the influence of regions with extensive strong LD, we used PLINK (ver. 1.90) with parameters -indep-pairphase 100 10 0.2 to generate an LD-pruned SNP dataset [51]. Finally, a subset of 6 222 538 SNPs were retained for analysis of phylogenetic and population structure. To evaluate the relatedness between individuals, the pairwise identity-by-state (IBS) genetic matrix was computed using PLINK with the parameter -distance 1-ibs flat-missing. Utilizing the distance matrix, a neighbor-joining phylogenetic tree was constructed using MEGA (ver. 6.0) [52]. Additionally, a PCA was constructed using PLINK with parameter —pca to further explore the population structure.

For the estimation and comparison of genetic diversity across populations of *C. gigantea* and *C. duclouxiana*, we calculated the average pairwise nucleotide diversity (π) using VCFtools (ver. 0.1.17) with 100-kb sliding windows in 10-kb steps [53]. Individual whole-genome heterozygosity was also determined using VCFtools with parameter —het. To further assess the LD pattern within each species or lineages, we calculated the correlation coefficient (*r*^2^) between any two loci using the program PopLDdcay (ver. 3.41) with parameter –maxDist 1000 [54].

### Demography inference

SMC++ (ver. 1.15.4) was used to infer population demography [55] based on neutral regions (excluding sites within 5-kb gene regions). Due to the linear scalability of computational and memory requirements with the total analyzed sequence length in SMC++, it is generally advisable to perform computations on a relatively small number of individuals (https://github.com/popgenmethods/smcpp#frequently-asked-questions). For each population of *C. gigantea* and *C. duclouxiana*, we therefore down-sampled to five (4 times) randomly selected individuals. The mutation rate (*μ*) was assumed to be 7.0 × 10^−9^ and the generation time (*g*) was assumed to be 50 years [56]. To further validate the demographic history, we also employed Stairway Plot (ver. 2) to infer *N*_e_ based on the folded site frequency spectrum (SFS) for each species [57]. We employed 200 bootstraps to generate median estimations and calculate a 95% confidence interval (CI). Furthermore, we used GONE to infer recent changes in *N*_e_ [25]. We conducted 40 replicate analyses, with each analysis involving the random sampling of 50 000 SNPs from each chromosome. We focused on *N*_e_ changes within 200 generations, a time interval deemed reliable according to the User’s Guide of GONE.

### Genetic load and deleterious mutations

We estimated genetic load in *C. gigantea* and *C. duclouxiana* using two approaches. First, we computed the genetic diversity of 0-fold and 4-fold degenerate sites for each sample. The identification of 0-fold and 4-fold degenerate sites was performed using a Python script (https://github.com/hui-liu/Degeneracy). This process involves iterating across all four possible bases at each site along with a transcript. To assess the genomic extent of inbreeding, genome-wide ROHs were obtained using BCFtools (ver. 1.9) with default parameters [58]. ROHs longer than 100 kb were retained. Individual inbreeding levels were evaluated using *F*_ROH_, which quantifies the fraction of the genome covered by ROHs [13].

Second, we used SnpEff (ver. 5.0) to predict the impacts of SNPs on genes or proteins [59]. The variants were classified into three categories: (i) LoF, denoting those with high impact on transcription and translation, such as stop codon gain/loss and start codon loss; (ii) missense; and (iii) synonymous. In total, we identified 482 347 mutations. Missense SNPs were further divided into non-synonymous deleterious (SIFT score <0.05) and non-synonymous tolerated (SIFT score ≥0.05) categories, determined by the SIFT score generated with the SIFT 4G (ver. 6.2.1) software [60]. The UniRef90 protein database was employed to search for homologous sequences. Sites labeled as ‘NA’ and those classified as low confidence (85 364 mutations) were excluded. At each SNP position, we utilized est-sfs to determine the derived and ancestral allelic states, leveraging *J. microsperma* and *C. chengiana* as outgroups [61]. We further counted the number of LoF and deleterious variant sites for all derived alleles (the total number of derived alleles is calculated as twice the count for the homozygous genotype plus the count for the heterozygous genotype) occurring in ROH and outside-ROH regions for every individual. These counts were then standardized by the number of derived synonymous sites in the same genomic region.

### Prediction of the number of derived deleterious alleles

To further test the hypothesis of purging deleterious mutations in the *C. gigantea* populations, we performed theoretical predictions of the number of derived deleterious alleles. We followed the approach of Kleinman-Ruiz *et al*. [62], which is based on a model developed by García-Dorado [63, 64]. The model initially assumes the presence of an ancestral population characterized by a very large effective size (*N*_anc_), which approaches the mutation–selection–drift (MSD) equilibrium and has a haploid-derived allele number. Subsequently, as effective population size undergoes successive reductions to *N*_new_ over multiple generations, the model can predict the total number of segregating and fixed deleterious mutations, including those segregating within the ancestral population and those originating from ongoing mutation as the population approaches a new MSD equilibrium (see details in Supplementary Data).

We counted derived mutations for different combinations of selection coefficients (*s*) and dominance coefficients (*h*). Predictions were generated from weakly deleterious (*s* = 0.001), mildly deleterious (*s* = 0.01), and strongly deleterious (*s* = 0.1) selection coefficients. To avoid introducing a large hidden burden into large populations by assuming *h* = 0 and thereby possibly exaggerating the contribution of purging to the changes of overall derived counts, we used *h* = 0.05 to predict the highly recessive case. For the sake of symmetry, we also used *h* = 0.25 and 0.45 to predict partially recessive and roughly additive cases.

## Acknowledgements

This work was financially supported by the National Natural Science Foundation of China (Grant/Award Number U20A2080), the Second Tibetan Plateau Scientific Expedition and Research (STEP) program (Grant/Award Number 2019QZKK05020110), the Sichuan Science and Technology Program (Grant/Award Number 2023NSFSC0186), the Fundamental Research Funds for the Central Universities of Sichuan University (Grant/Award Numbers SCU2021D006 and SCU2022D003), and the Institutional Research Fund from Sichuan University (2021SCUNL102). We thank Ruth Shaw for constructive comments and Aurora García-Dorado for providing a script to predict derived deleterious mutations.

## Author contributions

K.M. and J.Liu designed the research, J.Li, Y.W., J.Luo, H.Y., S.T., and T.J. conducted field surveys and collected samples, Y.W., Y.Y., Z.H., J.Li, J.K., D.W., and S.W. performed data analyses, Y.W., Y.Y., Z.H., J.Luo, and K.M. wrote the draft, all authors read and revised the manuscript, and S.S.R., Y.W., K.M., and J.Liu finalized the manuscript.

## Data availability

The *C. gigantea* genome sequences and newly generated whole-genome sequencing data of the samples produced in this study have been deposited in the National Genomics Data Center (NGDC) with the accession numbers GWHDOOJ00000000 and CRA009774, respectively. The annotation gff3 file has been deposited in Figshare (https://figshare.com/articles/dataset/Cupressus_gigantea_genome_annotation/25264894).

## Code availability

The code used in this study is available at https://github.com/Wennie-s/Tibetan-cypress-population-genomics.

## Conflict of interest

The authors declare no competing interests.

## Supplementary data

Supplementary data are available at *Horticulture Research* online.

## Supplementary Material

Web_Material_uhae108
