# Supplementary material for: Efficient purging of deleterious mutations contributes to the survival of a rare conifer
Source: Hortic Res. 2024 Apr 11;11(6):uhae108. doi: 10.1093/hr/uhae108 (PMC11179848; doi:10.1093/hr/uhae108)
Supplement: Web_Material_uhae108 [file web_material_uhae108.pdf]

Supplementary Information for

Efficient purging of deleterious mutations contributes to the survival of a rare conifer

Yi Wang, Yongzhi Yang, Zhitong Han, Jialiang Li, Jian Luo, Heng Yang, Jingge Kuang, Dayu Wu, Shiyang Wang, Sonam Tso, Tsam Ju, Jianquan Liu*, Susanne S. Renner*, Kangshang Mao*

*Correspondence:
maokangshan@scu.edu.cn (Kangshan Mao)
srenner@wustl.edu (Susanne S. Renner)
liujq@nwipb.ac.cn (Jianquan Liu)

The PDF file includes:

Supplementary figures: Figs. S1 to S16
Supplementary tables: Tables S1 to S13
Supplementary methods
Supplementary references

Supplementary figures

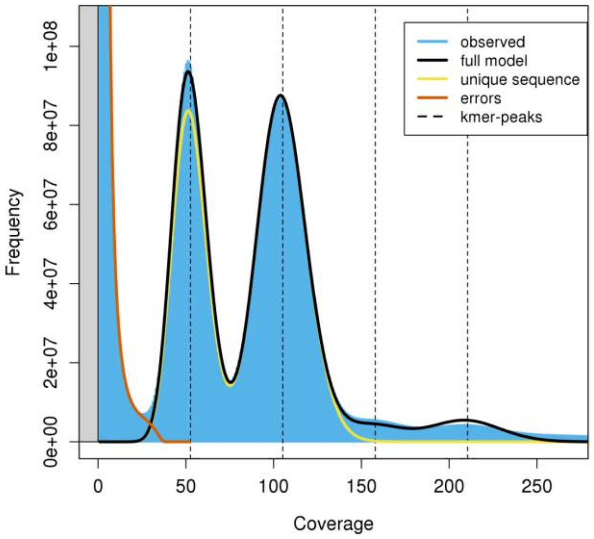

Fig. S1 | Distribution of 25-mer frequency in the *Cupressus gigantea* genome. A total of 1,380.98 Gb high-quality short-insert reads was used to generate the 25-mer depth distribution graph.

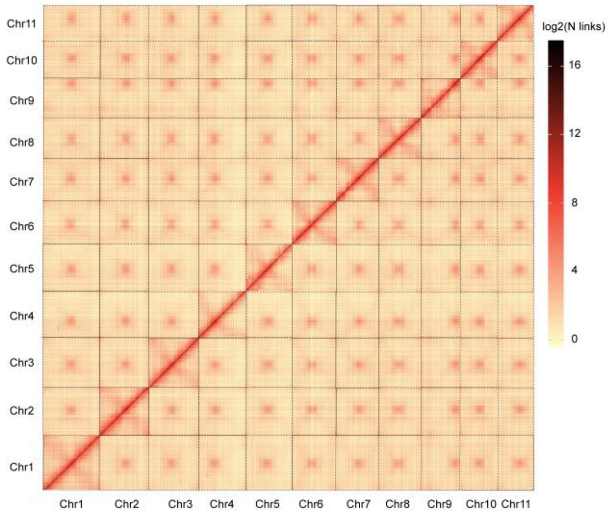

Fig. S2 | DNA Hi-C interactions of the 11 chromosomes. Each heat map shows a normalized contact matrix, with strong contacts in red and weak contacts in yellow. Black lines depict scaffold boundaries.

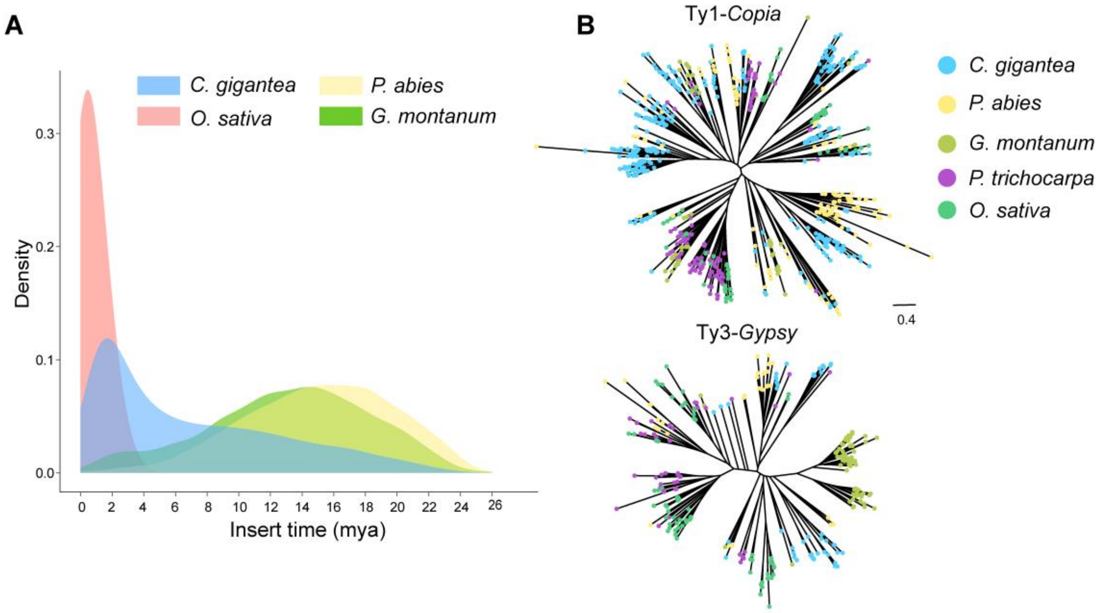

Fig. S3 | Analyses of LTR-RTs activity. **(A)** Estimation of LTR activity showing a burst in *Cupressus gigantea* less than two million years ago (Mya). **(B)** Heuristic neighbor-joining trees of full length Ty1-*Copia* and Ty3-*Gypsy* from *C. gigantea*, *Picea abies*, *Gnetum montanum*, *Populus trichocarpa*, *Oryza sativa*.

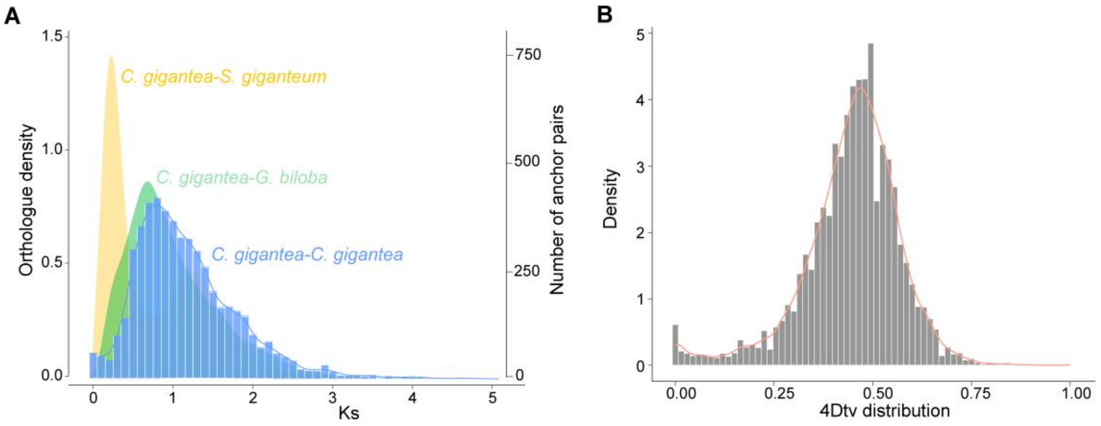

Fig. S4 | Genome duplication in *Cupressus gigantea*. **(A)** Distribution of synonymous substitution rates per gene (Ks) for the whole paranome of *C. gigantea.* Ks values revealed an ancient WGD event shared by *C. gigantea* and *G. biloba*. **(B)** Distribution of 4DTv site for the whole paranome of *C. gigantea*.

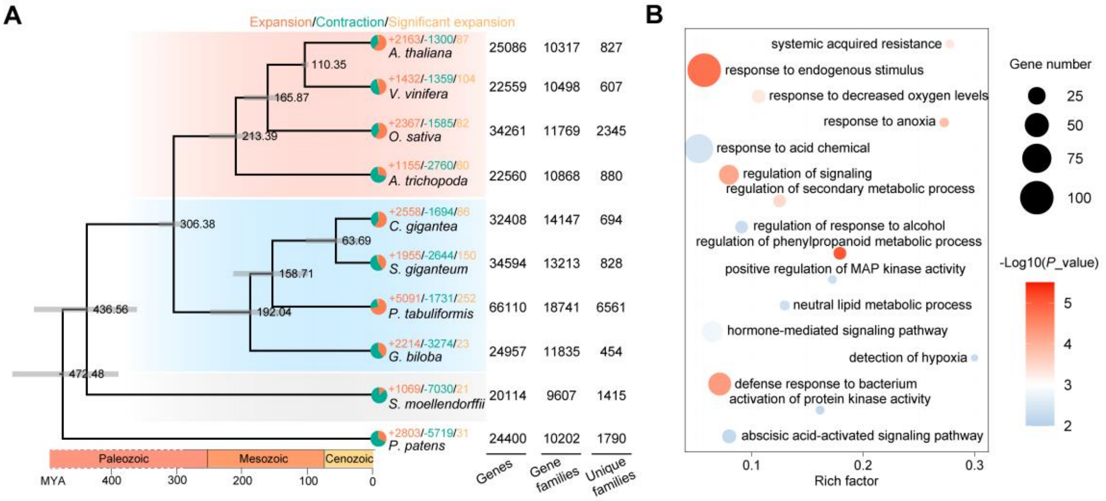

Fig. S5 | Gene family evolution of *Cupressus gigantea*. **(A)** Phylogenetic tree for *C. gigantea* and nine other gymnosperms and angiosperms. All bootstrap values are 100. The estimated divergence time (million years ago, Mya) is indicated at nodes; bars are 95% confidence intervals (CI). The number of gene families that expanded, contracted, or significant expanded is highlighted in orange, green, and yellow, respectively; the corresponding proportions among the total changes are shown using the same colours in the pie charts. **(B)** Function enrichment analysis of specific gene families in *C. gigantea*. The circle colour represents the statistical significance of enriched GO terms. The size of the circles is proportional to the number of genes in a GO term.

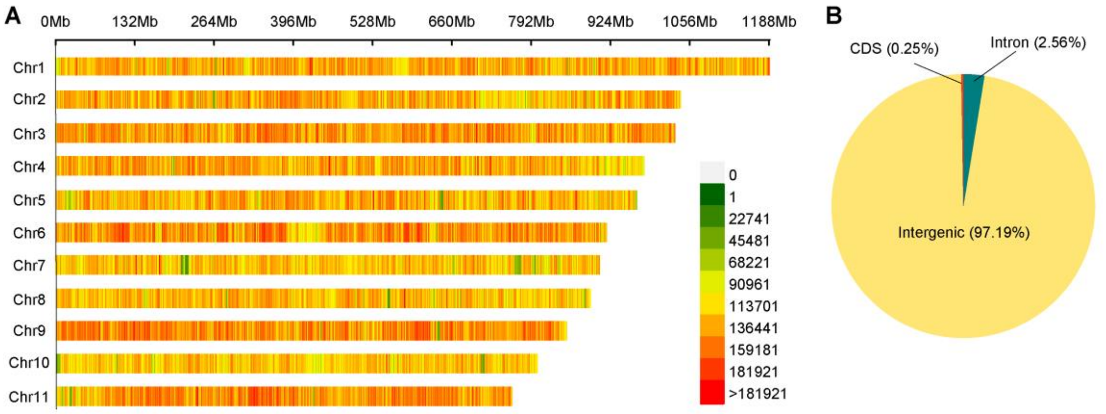

Fig. S6 | The density and distribution of SNPs in *Cupressus gigantea*. **(A)** SNPs density along chromosomes in 1 Mb windows. Colours indicate the number of SNPs per 1 Mb window. **(B)** Distribution of function annotation for SNPs.

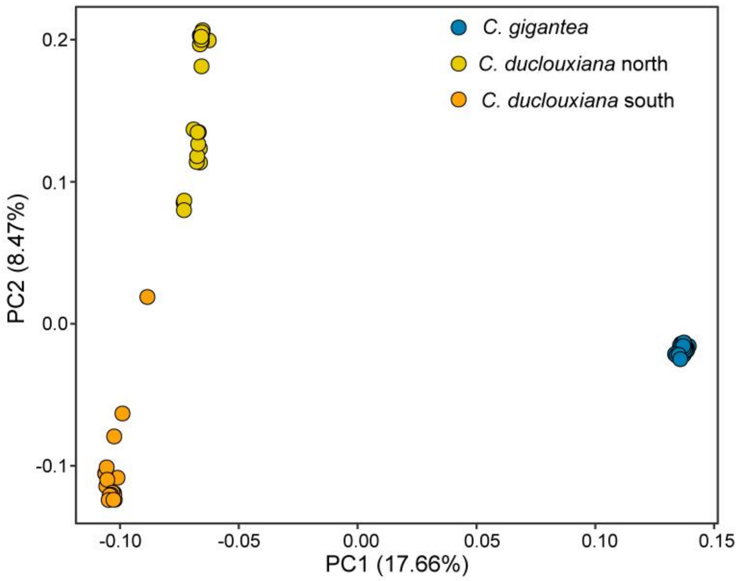

Fig. S7 | Principal component analysis of 83 accessions of *Cupressus gigantea* and *C. duclouxiana*, showing the first two components. PC1, representing 17.66% of the total variation, separates the *C. gigantea* from *C. duclouxiana*, whereas PC2, representing 8.47% of the total variation, distinguishes northern and southern *C. duclouxiana*.

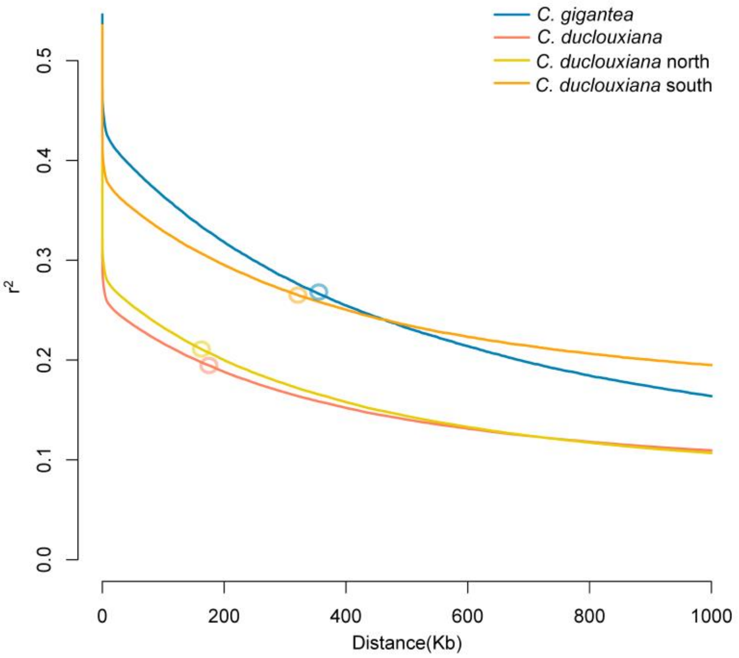

Fig. S8 | Linkage disequilibrium (LD) decay measured by r^2 in *Cupressus gigantea* and *C. duclouxiana*. Open circles denote distances where the r^2 correlation coefficient reduces to half of its maximum (approximately 350 kb for *C. gigantea* and approximately 185 kb for *C. duclouxiana*).

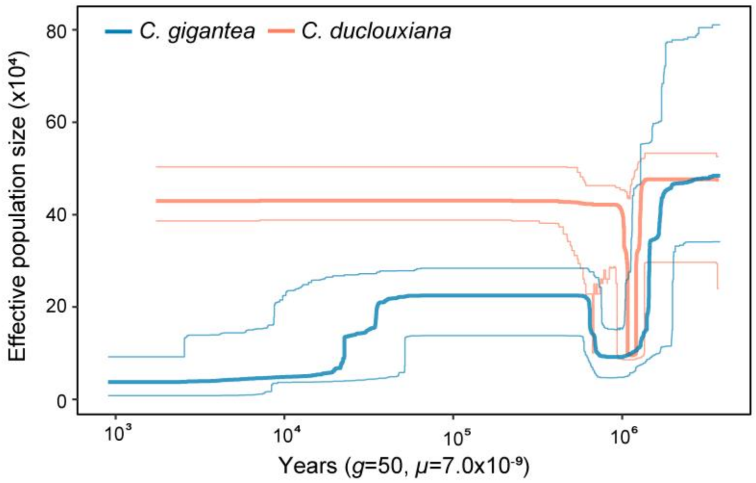

Fig. S9 | Effective population size (N_e) of *Cupressus gigantea* and *C. duclouxiana* from Stairway Plot. The time scale on the *x* axis is calculated assuming a mutation rate per generation (μ) of 7.0×10^{-9} and a generation time (g) of 50 years. Thick lines represent the median, and thin light lines represent the 95% pseudo-CI defined by the 2.5% and 97.5% estimations from the SFS analysis.

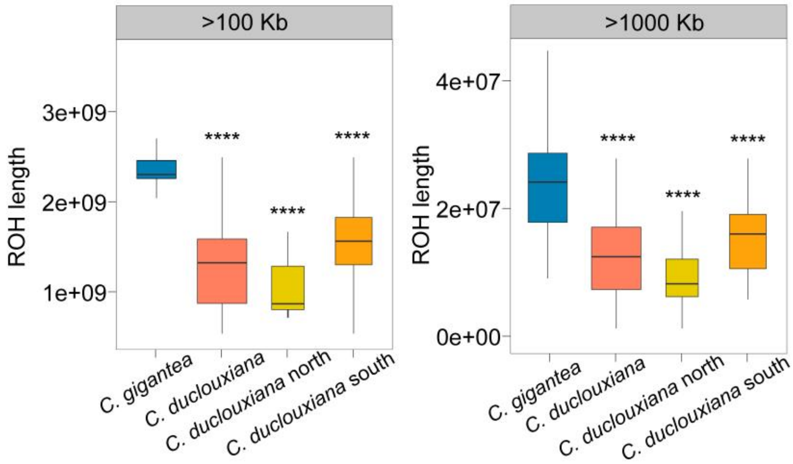

Fig. S10 | Comparison of ROH length. *P* values for comparisons were obtained from Welch's t-tests, and asterisks indicate the degree of significance (****, $P < 0.0001$, comparisons were conducted between *Cupressus gigantea* and *C. duclouxiana*, the latter either as a single entity or instead separated into its northern and southern populations).

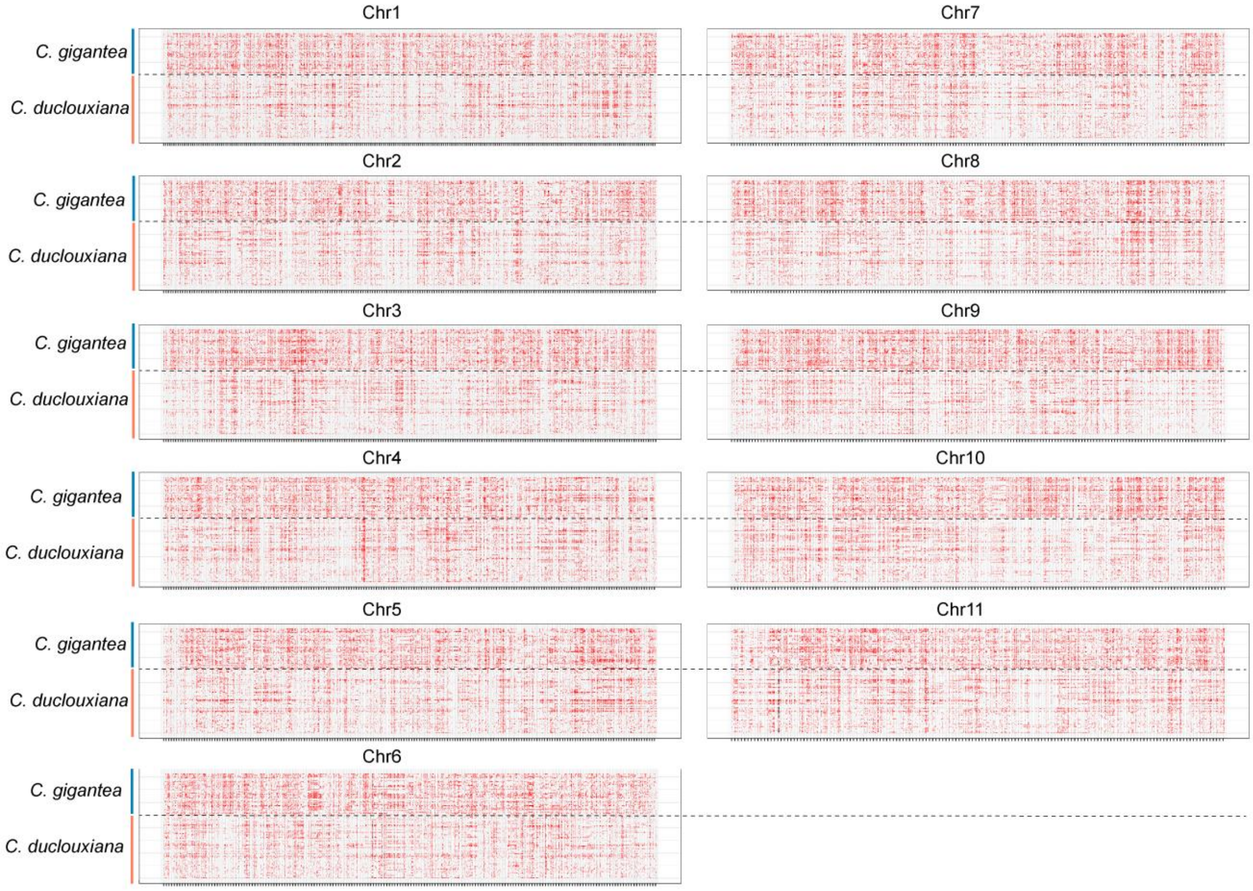

Fig. S11 | Distribution of ROH ≥ 100 kb and ROH ≥ 1000 kb along the 11 chromosomes for 32 *Cupressus gigantea* and 51 *C. duclouxiana* individuals. *C. gigantea*, top panel; *C. duclouxiana*, bottom panel; ROH ≥ 100 kb, red line, ROH ≥ 1000 kb, black line. Regions of ROH ≥ 1000 kb are extremely rare in both species.

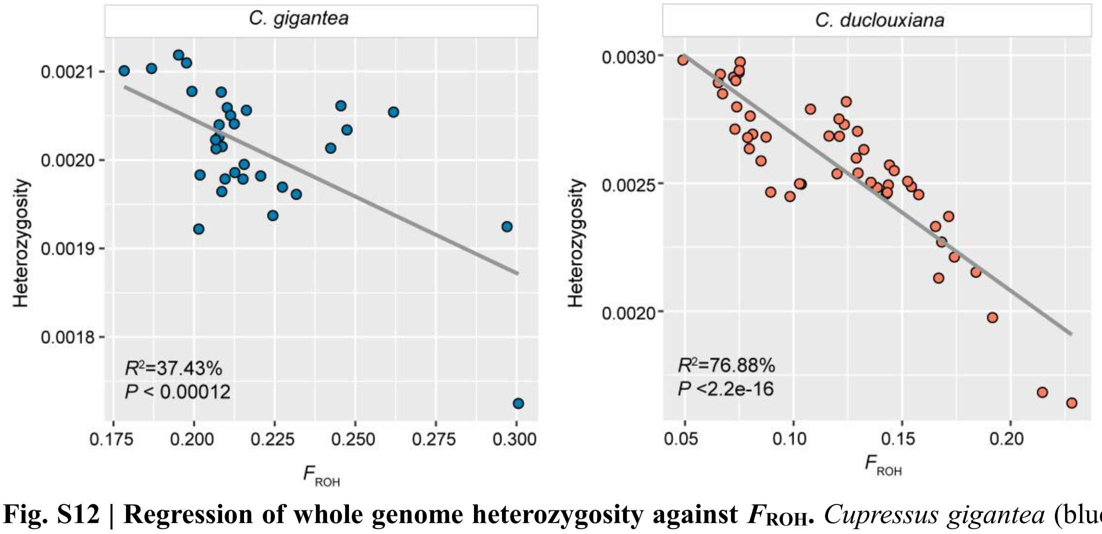

Fig. S12 | Regression of whole genome heterozygosity against F_{ROH}. *Cupressus gigantea* (blue circle), *C. duclouxiana* (orange circle).

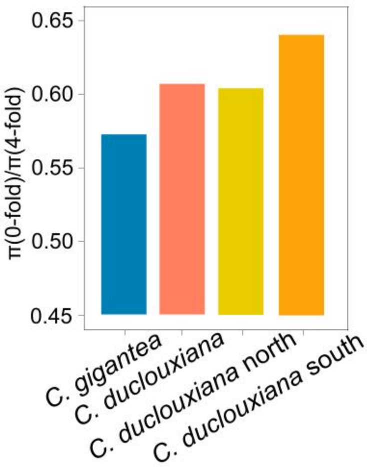

Fig. S13 | A comparison of π (0-fold degeneration variants) / π (4-fold degeneration variants) ratios in *Cupressus* species and populations.

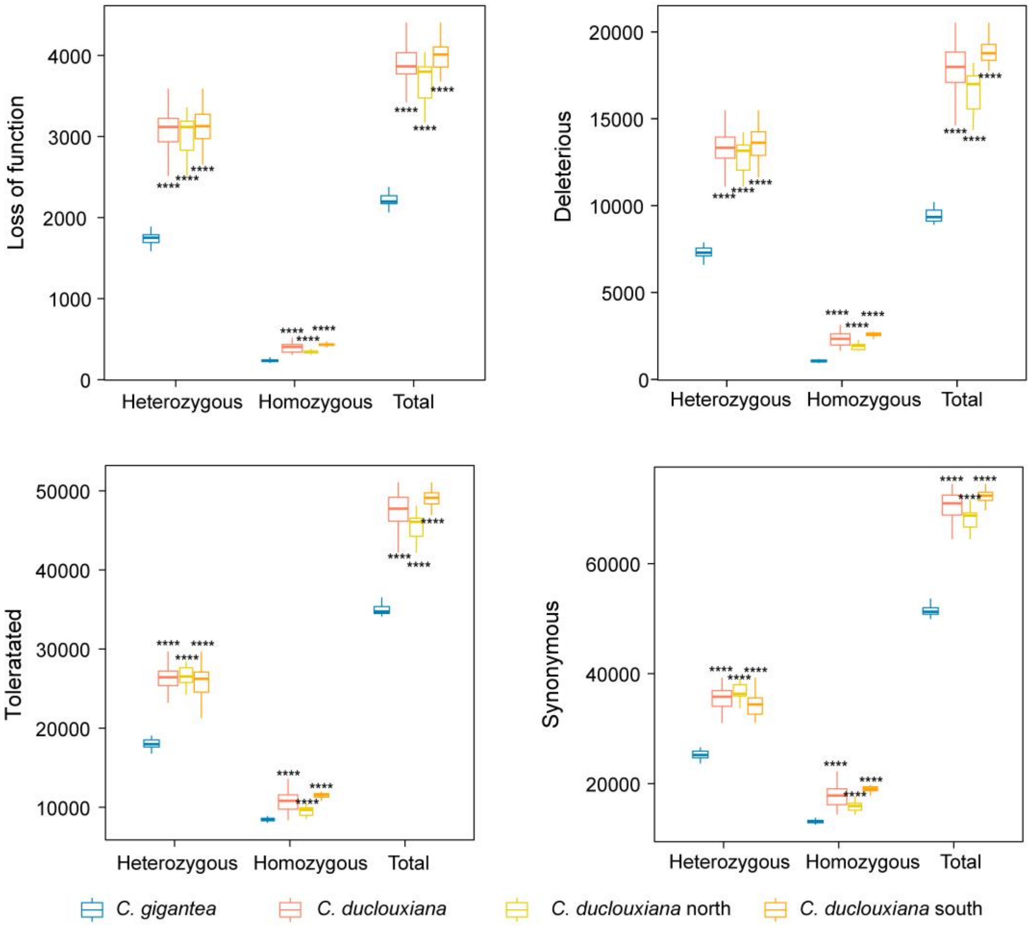

Fig. S14 | Number of derived variants in *Cupressus gigantea* and *C. duclouxiana*. The total number of derived alleles is based on counting each heterozygous genotype once and each homozygous genotype twice. *P* values for comparisons were obtained from Welch's t-tests, and asterisks indicate the degree of significance (****, $P < 0.0001$, comparisons were conducted between *Cupressus gigantea* and *C. duclouxiana*, the latter either as a single entity or instead separated into its northern and southern populations).

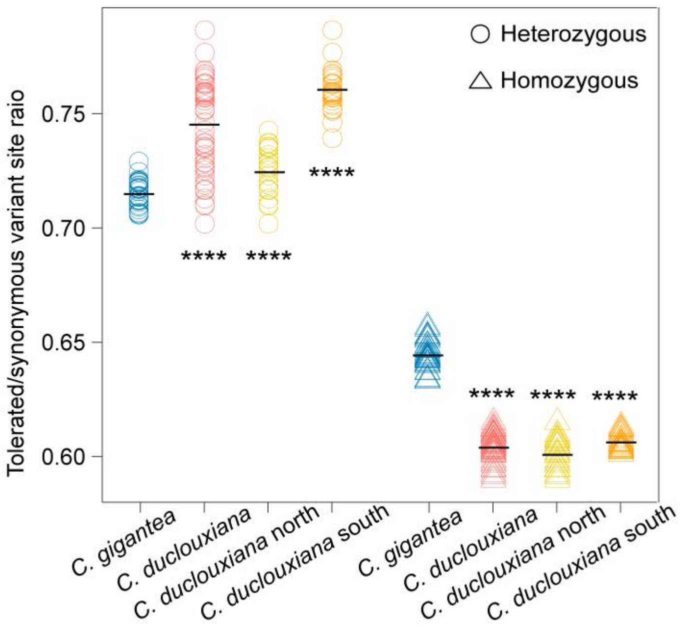

Fig. S15 | Ratio of derived tolerated variants to derived synonymous variants in heterozygous (circles) and homozygous (triangles) tracts per individual. Horizontal bars denote the average values. *P* values for the comparisons were obtained from Welch's t-tests, and asterisks indicate the degree of significance (****, $P < 0.0001$, comparisons were conducted between *Cupressus gigantea* and *C. duclouxiana*, the latter either as a single entity or instead separated into its northern and southern populations).

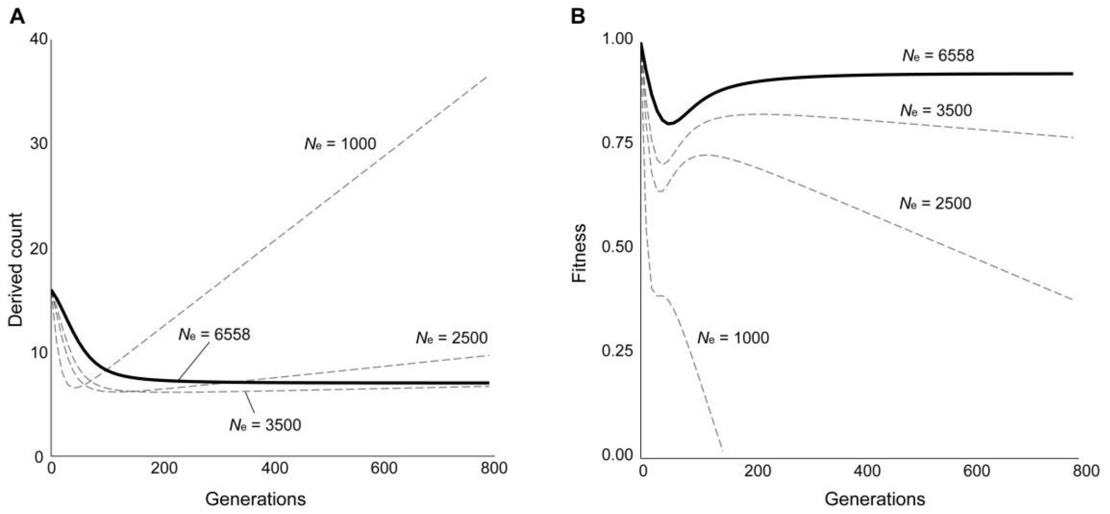

Fig. S16 | Predictions illustrating the relative effects of purging and drift in determining the evolution of derived alleles and fitness averages over time, with different effective population sizes (*Ne*) derived from a common population at the MSD balance with $N_e = 10^6$. (A) Evolution of derived alleles. **(B)** Evolution of average fitness. Assuming a haploid mutation rate of $\lambda = 1$, $s = 0.001$, $h = 0.05$

Supplementary tables

Supplementary Table S1 Survey statistics results of *Cupressus gigantea*.

K-mer	K-mer number	Genome size (bp)	Repeat (%)	Heterozygous ratio (%)	Used bases (bp)	Sequence depth (X)
19	1,213,759,768,722	10,261,083,156	77.97	1.22%	1,378,457,016,974	134.33
**25**	**1,158,239,221,094**	**10,380,627,249**	**60.90**	**1.17%**	**1,377,725,042,883**	**132.72**
31	1,102,712,863,674	10,379,605,346	51.92	1.06%	1,377,648,000,973	132.72

Note: Bold numbers indicate the genome size estimated based on 25-mer statistics.

Supplementary Table S2 Summary of *Cupressus gigantea* contig level assembly.

	Contig length (bp)	Number
N10	5,928,076	129
N20	4,022,479	358
N30	2,909,809	679
N40	2,200,668	1,110
N50	1,606,891	1,698
N60	1,110,845	2,513
N70	761,320	3,697
N80	462,796	5,543
N90	222,013	8,937
Short reads mapping rate	99.87%	

Note: N50 refers to the size above which 50% of the total length of the sequence assembly can be found.

Supplementary Table S3 Completeness of the assembly and annotated genes measured by Benchmarking Universal Single-Copy Orthologs (BUSCO).

Type	Assembly		Annotated genes	
	Number	Percentage (%)	Number	Percentage (%)
Complete BUSCOs	1296	80.3	1172	72.7
Complete and single-copy BUSCOs	1149	71.2	853	52.9
Complete and duplicated BUSCOs	147	9.1	319	19.8
Fragmented BUSCOs	169	10.5	261	16.2
Missing BUSCOs	149	9.2	181	11.1
Total	1614		1614	

Supplementary Table S4 Genome completeness estimated by RNA-seq evaluation.

Sample name	Total reads	Alignment reads	%	Unique hits	%	Multiple hits	%	Not alignment	%
Cg-1-leaf-1A	46412580	43503822	93.73	42001535	90.5	1502287	3.24	2908758	6.27
Cg-1-root-1B	46975694	38782351	82.56	37317147	79.44	1465204	3.12	8193343	17.44
Cg-1-stem1-1A	47000888	41029146	87.29	39398936	83.83	1630210	3.47	5971742	12.71
Cg-1-stem2-1A	47843732	40677924	85.02	39046566	81.61	1631358	3.41	7165808	14.98
5B-1A	41325842	38836113	93.98	37443148	90.6	1392965	3.37	2489729	6.02
5C-1A	47966426	45302585	94.45	43727459	91.16	1575126	3.28	2663841	5.55
5L-1A	47849192	43424104	90.75	41844527	87.45	1579577	3.3	4425088	9.25
5S-1A	46839824	43275255	92.39	41618941	88.85	1656314	3.54	3564569	7.61

Supplementary Table S5 Summary of transposable elements in the assembled *Cupressus gigantea* genome.

Type	Length(bp)	Rate (%)
**Class I:Retrotransposon**	5,889,709,114	53.94
**LTR-Retrotransposon**	5,228,927,338	47.89
LTR/Copia	2,414,919,795	22.12
LTR/Gypsy	2,793,845,138	25.59
LTR-other	20,162,405	0.18
**Non-LTR Retrotransposon**	660,781,776	6.05
SINE	116,886	0
LINE	660,664,890	6.05
**Class II:DNA Transposon**	653,447,204	5.98
EnSpm/CACTA	184,085,001	1.69
Harbinger	29,316,582	0.27
Helitron	41,967,124	0.38
MuDR	53,468,255	0.49
Tcl/Mariner	68,227,643	0.62
hAT	128,103,750	1.17
DNA-other	148,278,849	1.36
**Tandem repeat**	183,863,199	0.68
**Unclassified**	2,949,716,709	27.01
**Total content**	9,676,736,226	88.62
Genome Length	10,919,522,436	

Note: LTR, long terminal repeat transposable element; SINE, short interspersed nuclear elements; LINE, long interspersed nuclear elements.

Supplementary Table S6 Protein-coding genes predicted from the *Cupressus gigantea* assembly. Annotations were generated using *ab initio*-based, homology-based and RNA-seq-based approaches.

Type	Count	Max length (bp)	Min length (bp)	Median length(bp)	Average length (bp)
Gene	35,384	433,502	156	1,535.5	7897.27
mRNA	40,910	30,162	153	794	1191.01
CDS	40,910	14,193	150	651	908.88
Protein	-	-	-	217	302.96
Utr3	-	-	-	270	356.98
Utr5	-	-	-	130	233.87
Intron	-	-	-	187	2466.84

Supplementary Table S7 Functional annotation for the predicted genes.

Database	Number	Percent (%)
NR	31,152	88.04
Swiss-Prot	25,516	72.11
TAIR	28,809	81.42
msu	28,520	80.6
GO	12,787	36.14
KEGG	15,840	44.77
COG	27,566	77.91
eggNOG	27,566	77.91
Pfam	22,810	64.46
Annotation	31,306	88.48
Total	35,384	

Supplementary Table S8 Summary of gene family clustering between *Cupressus gigantea* and nine other plant species.

Species	Genes number	Genes in families	Unclustered genes	family number	Unique family genes	Shared families
Arabidopsis thaliana	27416	25086	2330	10317	3874	5004
Vitis vinifera	26346	22559	3787	10498	2137	
Oryza sativa	42006	34261	7745	11769	12164	
Amborella trichopoda	26846	22560	4286	10868	4397	
Cupressus gigantea	35384	32408	2976	14147	2680	
Sequoiadendron giganteum	37936	34594	3342	13213	4316	
Pinus tabuliformis	80464	66110	14354	18741	24545	
Ginkgo biloba	27814	24957	2857	11835	1639	
Selaginella moellendorffii	22285	20114	2171	9607	6176	
Physcomitrella patens	32926	24400	8526	10202	6434	

Supplementary Table S9 GO and KEGG enrichment of *Cupressus gigantea* expanded gene families

	Term	ID	Annotated	Significant	*P* value	Adjust *P* value
	cytokinesis by cell plate formation	GO:0000911	133	85	1.60E-14	1.00E-10
	mitotic cytokinetic process	GO:1902410	137	85	1.90E-13	3.00E-10
	(1->3)-beta-D-glucan catabolic process	GO:0006076	27	26	1.80E-12	1.50E-09
	beta-glucan catabolic process	GO:0051275	27	26	1.80E-12	1.50E-09
	flower morphogenesis	GO:0048439	30	25	7.30E-09	3.02E-06
	regulation of carbohydrate metabolic process	GO:0006109	69	45	9.80E-09	3.68E-06
	benzoate metabolic process	GO:0018874	24	21	2.20E-08	7.58E-06
	para-aminobenzoic acid metabolic process	GO:0046482	25	21	9.50E-08	2.76E-05
	regulation of 1-deoxy-D-xylulose-5-phosphate synthase activity	GO:1902395	14	14	1.00E-07	2.76E-05
	cytoskeleton-dependent cytokinesis	GO:0061640	183	92	1.00E-07	2.76E-05
	spindle organization	GO:0007051	50	34	1.40E-07	3.62E-05
	cell cycle process	GO:0022402	546	228	2.20E-07	5.05E-05
	cellular hyperosmotic salinity response	GO:0071475	24	20	2.50E-07	5.44E-05
	**cellular response to hydrogen peroxide**	**GO:0070301**	**36**	**26**	**6.80E-07**	**0.00012**
	**flavone biosynthetic process**	**GO:0051553**	**38**	**27**	**7.00E-07**	**0.00012**
	indolebutyric acid metabolic process	GO:0080024	25	20	8.90E-07	0.00014
	outer mitochondrial membrane organization	GO:0007008	12	12	1.00E-06	0.00014
	protein insertion into mitochondrial outer membrane	GO:0045040	12	12	1.00E-06	0.00014
	carpel morphogenesis	GO:0048445	12	12	1.00E-06	0.00014
	establishment of protein localization to mitochondrial membrane	GO:0090151	12	12	1.00E-06	0.00014
**GO terms**	cellular aldehyde metabolic process	GO:0006081	54	34	2.10E-06	0.00027
	monocarboxylic acid metabolic process	GO:0032787	521	214	2.20E-06	0.00028
	polysaccharide catabolic process	GO:0000272	104	55	5.40E-06	0.00063
	carbohydrate catabolic process	GO:0016052	156	76	5.90E-06	0.00066
	flower development	GO:0009908	570	229	6.40E-06	0.0007
	cell differentiation	GO:0030154	830	320	7.10E-06	0.00075
	**cellular response to osmotic stress**	**GO:0071470**	**72**	**41**	**7.80E-06**	**0.00081**
	mitotic spindle organization	GO:0007052	33	23	8.00E-06	0.00081
	reproductive shoot system development	GO:0090567	589	235	8.70E-06	0.00086
	**jasmonic acid biosynthetic process**	**GO:0009695**	**61**	**36**	**9.30E-06**	**0.00089**
	regulation of mitotic cell cycle phase transition	GO:1901990	42	27	1.40E-05	0.00132
	carbohydrate derivative metabolic process	GO:1901135	453	185	1.60E-05	0.00147
	establishment of protein localization to membrane	GO:0090150	34	23	1.70E-05	0.00149
	microtubule cytoskeleton organization involved in mitosis	GO:1902850	34	23	1.70E-05	0.00149
	specification of floral organ identity	GO:0010093	28	20	1.80E-05	0.00149
	cell wall organization	GO:0071555	165	78	1.80E-05	0.00149
	glucan catabolic process	GO:0009251	81	44	1.90E-05	0.00151
	regulation of cell shape	GO:0008360	45	28	2.30E-05	0.00179
	regulation of vegetative meristem growth	GO:0010083	17	14	2.40E-05	0.0018
	regulation of isopentenyl diphosphate biosynthetic process, methylerythritol 4-phosphate pathway	GO:0010322	17	14	2.40E-05	0.0018

cellular carbohydrate catabolic process	GO:0044275	101	52	2.60E-05	0.00192
response to karrikin	GO:0080167	207	93	3.80E-05	0.00276
mitochondrial membrane organization	GO:0007006	14	12	4.60E-05	0.00328
negative regulation of growth	GO:0045926	53	31	4.90E-05	0.00343
cellular localization	GO:0051641	528	208	7.10E-05	0.00473
regulation of phospholipid biosynthetic process	GO:0071071	18	14	7.60E-05	0.00491
arabinan metabolic process	GO:0031221	11	10	7.90E-05	0.00502
spindle assembly	GO:0051225	28	19	9.00E-05	0.00564
shade avoidance	GO:0009641	26	18	9.20E-05	0.00568
regulation of lipid localization	GO:1905952	8	8	0.0001	0.00599
carbohydrate derivative biosynthetic process	GO:1901137	282	119	0.00011	0.00623
small molecule metabolic process	GO:0044281	1253	455	0.00011	0.00623
nucleobase-containing small molecule metabolic process	GO:0055086	228	99	0.00011	0.00623
xylan catabolic process	GO:0045493	13	11	0.00013	0.00726
cellular carbohydrate metabolic process	GO:0044262	336	138	0.00014	0.00772
defense response by callose deposition	GO:0052542	58	32	0.00017	0.00925
carbohydrate homeostasis	GO:0033500	40	24	0.0002	0.01074
vegetative meristem growth	GO:0010448	19	14	0.00021	0.01085
cellular developmental process	GO:0048869	1056	386	0.00021	0.01085
positive gravitropism	GO:0009958	23	16	0.00021	0.01085
hemicellulose metabolic process	GO:0010410	38	23	0.00023	0.01146
L-phenylalanine catabolic process	GO:0006559	10	9	0.00023	0.01146
erythrose 4-phosphate/phosphoenolpyruvate family amino acid catabolic process	GO:1902222	10	9	0.00023	0.01146
establishment of organelle localization	GO:0051656	83	42	0.00025	0.0123
**cellular response to salt stress**	**GO:0071472**	**52**	**29**	**0.00027**	**0.01298**
beta-glucan metabolic process	GO:0051273	69	36	0.00031	0.01438
specification of carpel identity	GO:0010094	7	7	0.00032	0.01438
cellular sphingolipid homeostasis	GO:0090156	7	7	0.00032	0.01438
nucleoside phosphate metabolic process	GO:0006753	177	78	0.00033	0.01467
**salicylic acid metabolic process**	**GO:0009696**	**62**	**33**	**0.00034**	**0.01495**
regulation of small molecule metabolic process	GO:0062012	125	58	0.00038	0.0162
cell wall modification	GO:0042545	87	43	0.0004	0.01686
cellular catabolic process	GO:0044248	690	259	0.00042	0.01686
seed trichome differentiation	GO:0090376	14	11	0.00042	0.01686
**defense response by cell wall thickening**	**GO:0052482**	**53**	**29**	**0.00042**	**0.01686**
organelle assembly	GO:0070925	65	34	0.00043	0.01709
regulation of shoot system development	GO:0048831	310	126	0.00045	0.01725
callose localization	GO:0052545	70	36	0.00045	0.01725
attachment of spindle microtubules to kinetochore	GO:0008608	16	12	0.00046	0.01725
coenzyme A metabolic process	GO:0015936	16	12	0.00046	0.01725
plant ovule development	GO:0048481	90	44	0.00047	0.01725
cellular ketone metabolic process	GO:0042180	139	63	0.00048	0.01725
meristem determinacy	GO:0010022	18	13	0.00048	0.01725
floral meristem determinacy	GO:0010582	18	13	0.00048	0.01725

isopentenyl diphosphate biosynthetic process	GO:0019288	20	14	0.00049	0.01746
organic substance catabolic process	GO:1901575	681	255	0.00054	0.01908
plant-type cell wall organization	GO:0009664	86	42	0.00064	0.02221
arabinan catabolic process	GO:0031222	9	8	0.00065	0.02221
**regulation of proanthocyanidin biosynthetic process**	**GO:2000029**	**9**	**8**	**0.00065**	**0.02221**
shoot system morphogenesis	GO:0010016	307	124	0.00066	0.02236
gynoecium development	GO:0048467	117	54	0.00069	0.02319
**cell wall thickening**	**GO:0052386**	**64**	**33**	**0.00072**	**0.02334**
callose deposition in cell wall	GO:0052543	64	33	0.00072	0.02334
positive regulation of seed germination	GO:0010030	52	28	0.00074	0.02334
cellular glucan metabolic process	GO:0006073	181	78	0.00074	0.02334
protein localization to membrane	GO:0072657	45	25	0.00076	0.02334
obsolete movement of cell or subcellular component	GO:0006928	120	55	0.00077	0.02334
antibiotic biosynthetic process	GO:0017000	27	17	0.0008	0.02334
purine ribonucleotide biosynthetic process	GO:0009152	57	30	0.0008	0.02334
organelle localization	GO:0051640	87	42	0.00087	0.02334
organic hydroxy compound biosynthetic process	GO:1901617	231	96	0.00087	0.02334
regulation of cell motility	GO:2000145	25	16	0.00088	0.02334
cell wall polysaccharide metabolic process	GO:0010383	82	40	0.00088	0.02334
maintenance of floral organ identity	GO:0048497	11	9	0.0009	0.02334
inorganic cation import across plasma membrane	GO:0098659	11	9	0.0009	0.02334
nucleoside phosphate biosynthetic process	GO:1901293	113	52	0.00094	0.02334
mitotic cell cycle phase transition	GO:0044772	60	31	0.00099	0.02334
regulation of glycolytic process	GO:0006110	6	6	0.00101	0.02334
regulation of nucleotide catabolic process	GO:0030811	6	6	0.00101	0.02334
regulation of lipid transport	GO:0032368	6	6	0.00101	0.02334
negative regulation of stress-activated MAPK cascade	GO:0032873	6	6	0.00101	0.02334
formaldehyde metabolic process	GO:0046292	6	6	0.00101	0.02334
negative regulation of JNK cascade	GO:0046329	6	6	0.00101	0.02334
negative regulation of gene silencing by RNA	GO:0060967	6	6	0.00101	0.02334
testosterone biosynthetic process	GO:0061370	6	6	0.00101	0.02334
obsolete regulation of glycolytic process by regulation of transcription from RNA polymerase II promoter	GO:0072361	6	6	0.00101	0.02334
obsolete regulation of lipid transport by regulation of transcription from RNA polymerase II promoter	GO:0072367	6	6	0.00101	0.02334
obsolete regulation of carbohydrate catabolic process by regulation of transcription from RNA polymerase II promoter	GO:0098679	6	6	0.00101	0.02334
negative regulation of miRNA maturation	GO:1903799	6	6	0.00101	0.02334
regulation of ATP biosynthetic process	GO:2001169	6	6	0.00101	0.02334
metaphase/anaphase transition of mitotic cell cycle	GO:0007091	21	14	0.00103	0.02334
regulation of mitotic sister chromatid separation	GO:0010965	21	14	0.00103	0.02334
regulation of developmental vegetative growth	GO:1905613	21	14	0.00103	0.02334
regulation of chromosome separation	GO:1905818	21	14	0.00103	0.02334

response to molecule of fungal origin	GO:0002238	13	10	0.00105	0.02334
lactate metabolic process	GO:0006089	13	10	0.00105	0.02334
fruit morphogenesis	GO:0048530	13	10	0.00105	0.02334
methylglyoxal catabolic process to lactate	GO:0061727	13	10	0.00105	0.02334
mitotic spindle assembly	GO:0090307	13	10	0.00105	0.02334
L-phenylalanine metabolic process	GO:0006558	15	11	0.00112	0.02412
establishment of chromosome localization	GO:0051303	15	11	0.00112	0.02412
erythrose 4-phosphate/phosphoenolpyruvate family amino acid metabolic process	GO:1902221	15	11	0.00112	0.02412
protein targeting to mitochondrion	GO:0006626	17	12	0.00112	0.02412
external encapsulating structure organization	GO:0045229	211	88	0.00123	0.02608
plant epidermal cell differentiation	GO:0090627	211	88	0.00123	0.02608
phenol-containing compound metabolic process	GO:0018958	96	45	0.00126	0.02658
pyruvate metabolic process	GO:0006090	51	27	0.00128	0.02672
regulation of cell cycle phase transition	GO:1901987	51	27	0.00128	0.02672
**cellular response to reactive oxygen species**	**GO:0034614**	**91**	**43**	**0.00131**	**0.02721**
**cellular response to oxidative stress**	**GO:0034599**	**125**	**56**	**0.00135**	**0.02777**
organophosphate metabolic process	GO:0019637	395	153	0.00146	0.02988
mitotic nuclear division	GO:0140014	84	40	0.00159	0.0322
glyceraldehyde-3-phosphate metabolic process	GO:0019682	26	16	0.00161	0.0322
xylan metabolic process	GO:0045491	26	16	0.00161	0.0322
obsolete regulation of cellular component movement	GO:0051270	26	16	0.00161	0.0322
nucleotide biosynthetic process	GO:0009165	110	50	0.00162	0.0322
indole-containing compound metabolic process	GO:0042430	87	41	0.00179	0.03524
**flavonoid biosynthetic process**	**GO:0009813**	**129**	**57**	**0.00182**	**0.03566**
regulation of mitotic nuclear division	GO:0007088	33	19	0.00184	0.03588
catabolic process	GO:0009056	754	275	0.00204	0.03941
floral whorl development	GO:0048438	217	89	0.00212	0.04076
monocarboxylic acid biosynthetic process	GO:0072330	273	109	0.00215	0.04115
cell wall organization or biogenesis	GO:0071554	324	127	0.00217	0.04134
nitrate transport	GO:0015706	50	26	0.0022	0.04172
programmed cell death in response to reactive oxygen species	GO:0097468	20	13	0.00221	0.04172
negative regulation of developmental process	GO:0051093	187	78	0.00225	0.04228
establishment of localization in cell	GO:0051649	376	145	0.00235	0.04242
cGMP biosynthetic process	GO:0006182	10	8	0.00235	0.04242
cyclic nucleotide metabolic process	GO:0009187	10	8	0.00235	0.04242
**histone H3-K9 demethylation**	**GO:0033169**	**10**	**8**	**0.00235**	**0.04242**
protein homotrimerization	GO:0070207	10	8	0.00235	0.04242
nucleoside bisphosphate biosynthetic process	GO:0033866	18	12	0.0024	0.04276
ribonucleoside bisphosphate biosynthetic process	GO:0034030	18	12	0.0024	0.04276
**cold acclimation**	**GO:0009631**	**43**	**23**	**0.00241**	**0.04276**
pectin biosynthetic process	GO:0045489	29	17	0.00245	0.04328
aromatic amino acid family metabolic process	GO:0009072	78	37	0.00254	0.04426
ameboidal-type cell migration	GO:0001667	16	11	0.00255	0.04426
methionine metabolic process	GO:0006555	16	11	0.00255	0.04426

	**osmosensory signaling pathway**	**GO:0007231**	**12**	**9**	**0.00258**	**0.04426**
	positive regulation of nuclear division	GO:0051785	12	9	0.00258	0.04426
	carboxylic acid metabolic process	GO:0019752	828	299	0.00258	0.04426
	response to glucose	GO:0009749	63	31	0.00272	0.04627
	response to symbiont	GO:0009608	41	22	0.00283	0.04775
	glycosyl compound biosynthetic process	GO:1901659	81	38	0.00286	0.04803
	developmental process involved in reproduction	GO:0003006	1756	606	0.00287	0.04803
	auxin metabolic process	GO:0009850	71	34	0.00307	0.04999
	UDP-N-acetylglucosamine metabolic process	GO:0006047	5	5	0.00318	0.04999
	nucleoside triphosphate catabolic process	GO:0009143	5	5	0.00318	0.04999
	UDP-rhamnose biosynthetic process	GO:0010253	5	5	0.00318	0.04999
	myo-inositol transport	GO:0015798	5	5	0.00318	0.04999
	floral whorl structural organization	GO:0048459	5	5	0.00318	0.04999
	bundle sheath cell fate specification	GO:0090610	5	5	0.00318	0.04999
	egative regulation of proteasomal protein catabolic process	GO:1901799	5	5	0.00318	0.04999
	03010 Ribosome	-	406	205	8.88E-16	1.94E-13
	00380 Tryptophan metabolism	-	149	86	3.56E-11	2.59E-09
	04121 Ubiquitin system	-	787	327	9.86E-10	5.38E-08
	00966 Glucosinolate biosynthesis	-	50	35	2.71E-08	1.18E-06
	00052 Galactose metabolism	-	117	64	1.79E-07	6.50E-06
	03013 Nucleocytoplasmic transport	-	267	124	2.26E-07	7.04E-06
	01003 Glycosyltransferases	-	404	174	6.04E-07	1.65E-05
	B 09122 Translation	-	985	381	6.67E-07	1.62E-05
	00997 Biosynthesis of various secondary metabolites-part 3	-	37	26	1.53E-06	3.33E-05
	00908 Zeatin biosynthesis	-	83	47	2.04E-06	4.05E-05
**KEGG terms**	**04075 Plant hormone signal transduction**	**-**	**294**	**127**	**1.62E-05**	**2.95E-04**
	B 09110 Biosynthesis of other secondary metabolites	-	608	239	2.44E-05	4.09E-04
	**00906 Carotenoid biosynthesis**	**-**	**75**	**41**	**2.85E-05**	**4.44E-04**
	04626 Plant-pathogen interaction	-	355	147	5.13E-05	7.46E-04
	A09150 Organismal Systems	-	421	168	1.59E-04	0.00216
	**B 09159 Environmental adaptation**	**-**	**421**	**168**	**1.59E-04**	**0.00216**
	00901 Indole alkaloid biosynthesis	-	10	9	2.24E-04	0.00271
	**00199 Cytochrome P450**	**-**	**350**	**140**	**4.77E-04**	**0.0052**
	99996 General function prediction only	-	25	16	8.55E-04	0.00887
	00260 Glycine, serine and threonine metabolism	-	181	77	0.00119	0.01177
	00600 Sphingolipid metabolism	-	83	39	0.00235	0.02228
	00360 Phenylalanine metabolism	-	80	37	0.00419	0.03805

Note: GO and KEGG terms in bold may be related to the adaptation of *C. gigantea* to high altitude.

Supplementary Table S10 GO and KEGG enrichment of *Cupressus gigantea* significantly expanded gene families

	Term	ID	Annotated	Significant	*P* value	Adjust *P* value
	cytokinesis by cell plate formation	GO:0000911	133	60	< 1e-30	0
	mitotic cytokinetic process	GO:1902410	137	60	< 1e-30	0
	cytoskeleton-dependent cytokinesis	GO:0061640	183	60	< 1e-30	0
	mitotic cell cycle process	GO:1903047	280	60	< 1e-30	0
	cell division	GO:0051301	392	60	8.60E-30	0
	response to karrikin	GO:0080167	207	40	9.20E-24	0
	cell cycle process	GO:0022402	546	60	5.60E-22	0
	protein folding	GO:0006457	44	18	1.30E-17	0
	oxylipin metabolic process	GO:0031407	24	14	9.20E-17	0
	regulation of 1-deoxy-D-xylulose-5-phosphate synthase activity	GO:1902395	14	11	1.20E-15	0
	lipid metabolic process	GO:0006629	872	64	2.00E-14	0
	egulation of isopentenyl diphosphate biosynthetic process, methylerythritol 4-phosphate pathway	GO:0010322	17	11	3.90E-14	0
	regulation of phospholipid biosynthetic process	GO:0071071	18	11	9.90E-14	0
	syncytium formation	GO:0006949	22	11	2.00E-12	1.00E-10
	isopentenyl diphosphate biosynthetic process	GO:0009240	25	11	1.20E-11	4.00E-10
	chaperone-mediated protein folding	GO:0061077	25	11	1.20E-11	4.00E-10
	glyceraldehyde-3-phosphate metabolic process	GO:0019682	26	11	2.00E-11	6.00E-10
	**response to stress**	**GO:0006950**	**3405**	**142**	**3.20E-11**	**9.00E-10**
	monocarboxylic acid metabolic process	GO:0032787	521	42	7.00E-11	1.80E-09
	**flavonol metabolic process**	**GO:0051554**	**37**	**12**	**9.60E-11**	**2.20E-09**
**GO Term**	**response to water deprivation**	**GO:0009414**	**486**	**40**	**1.10E-10**	**2.50E-09**
	response to water	GO:0009415	489	40	1.40E-10	2.70E-09
	**response to osmotic stress**	**GO:0006970**	**788**	**53**	**1.60E-10**	**3.00E-09**
	fatty acid omega-oxidation	GO:0010430	49	13	2.60E-10	4.70E-09
	defense response to other organism	GO:0098542	1112	65	3.50E-10	6.10E-09
	fatty acid metabolic process	GO:0006631	266	28	3.80E-10	6.40E-09
	**jasmonic acid biosynthetic process**	**GO:0009695**	**61**	**14**	**4.40E-10**	**7.20E-09**
	**response to salt stress**	**GO:0009651**	**673**	**47**	**6.00E-10**	**9.50E-09**
	**response to external biotic stimulus**	**GO:0043207**	**1499**	**78**	**9.30E-10**	**1.38E-08**
	response to other organism	GO:0051707	1499	78	9.30E-10	1.38E-08
	**response to biotic stimulus**	**GO:0009607**	**1503**	**78**	**1.10E-09**	**1.59E-08**
	oxidation-reduction process	GO:0055114	795	51	2.00E-09	2.73E-08
	saponin metabolic process	GO:0016134	17	8	4.30E-09	5.41E-08
	glycoside biosynthetic process	GO:0016138	17	8	4.30E-09	5.41E-08
	**response to abiotic stimulus**	**GO:0009628**	**2377**	**105**	**4.50E-09**	**5.52E-08**
	response to wounding	GO:0009611	377	32	4.70E-09	5.63E-08
	**regulation of isoprenoid metabolic process**	**GO:0019747**	**42**	**11**	**7.60E-09**	**8.79E-08**
	defense response to bacterium	GO:0042742	631	43	7.70E-09	8.79E-08
	regulation of cellular carbohydrate metabolic process	GO:0010675	46	11	2.10E-08	2.34E-07
	cellular lipid metabolic process	GO:0044255	758	47	2.80E-08	3.06E-07
	glycoside metabolic process	GO:0016137	21	8	3.30E-08	3.52E-07
	**triterpenoid metabolic process**	**GO:0006722**	**22**	**8**	**5.10E-08**	**5.33E-07**

pyruvate metabolic process	GO:0006090	51	11	6.80E-08	6.96E-07
**defense response**	**GO:0006952**	**1272**	**65**	**7.80E-08**	**7.82E-07**
steroid metabolic process	GO:0008202	134	17	8.20E-08	8.05E-07
cellular aldehyde metabolic process	GO:0006081	54	11	1.30E-07	1.25E-06
regulation of lipid biosynthetic process	GO:0046890	57	11	2.30E-07	2.17E-06
response to bacterium	GO:0009617	717	43	3.00E-07	2.78E-06
fatty acid oxidation	GO:0019395	89	13	5.50E-07	5.00E-06
regulation of carbohydrate metabolic process	GO:0006109	69	11	1.70E-06	1.52E-05
multi-organism process	GO:0051704	2032	86	1.80E-06	1.58E-05
**cellular response to hypoxia**	**GO:0071456**	**17**	**6**	**3.20E-06**	**2.71E-05**
regulation of cellular ketone metabolic process	GO:0010565	88	12	3.20E-06	2.71E-05
lipid oxidation	GO:0034440	104	13	3.40E-06	2.83E-05
xylem and phloem pattern formation	GO:0010051	139	15	3.90E-06	3.19E-05
cell-cell signaling	GO:0007267	107	13	4.60E-06	3.70E-05
defense response to Gram-negative bacterium	GO:0050829	21	6	1.30E-05	0.000103
regulation of lipid metabolic process	GO:0019216	90	11	2.40E-05	0.000187
**cellular response to decreased oxygen levels**	**GO:0036294**	**24**	**6**	**2.90E-05**	**0.000216**
**cellular response to oxygen levels**	**GO:0071453**	**24**	**6**	**2.90E-05**	**0.000216**
carboxylic acid metabolic process	GO:0019752	828	42	2.90E-05	0.000216
phospholipid biosynthetic process	GO:0008654	95	11	4.00E-05	0.000293
**response to external stimulus**	**GO:0009605**	**1976**	**79**	**4.80E-05**	**0.000347**
regulation of transferase activity	GO:0051338	98	11	5.30E-05	0.000377
regulation of small molecule metabolic process	GO:0062012	125	12	0.00011	0.000772
response to inorganic substance	GO:0010035	1244	54	0.00014	0.000968
**isoprenoid metabolic process**	**GO:0006720**	**279**	**19**	**0.00015**	**0.001009**
**flavonoid biosynthetic process**	**GO:0009813**	**129**	**12**	**0.00015**	**0.001009**
organic acid metabolic process	GO:0006082	892	42	0.00016	0.001047
oxoacid metabolic process	GO:0043436	892	42	0.00016	0.001047
**positive regulation of hydrogen peroxide metabolic process**	**GO:0010726**	**12**	**4**	**0.0002**	**0.001292**
cellular ketone metabolic process	GO:0042180	139	12	0.00031	0.001977
regulation of phosphate metabolic process	GO:0019220	125	11	0.00047	0.002921
**cutin biosynthetic process**	**GO:0010143**	**39**	**6**	**0.00051**	**0.00313**
response to acid chemical	GO:0001101	1410	57	0.00058	0.003516
lipid modification	GO:0030258	171	13	0.00061	0.003653
hyperosmotic response	GO:0006972	110	10	0.00065	0.003845
positive regulation of defense response to virus by host	GO:0002230	8	3	0.00093	0.005436
glycosyl compound biosynthetic process	GO:1901659	81	8	0.00132	0.007536
obsolete positive regulation of cofactor metabolic process	GO:0051194	20	4	0.00167	0.009425
cellular response to iron ion	GO:0071281	67	7	0.00189	0.010545
regulation of organic acid transport	GO:0032890	3	2	0.00206	0.01076
regulation of amine transport	GO:0051952	3	2	0.00206	0.01076
regulation of amino acid transport	GO:0051955	3	2	0.00206	0.01076
pollen germination	GO:0009846	87	8	0.00209	0.010802
pigment biosynthetic process	GO:0046148	177	12	0.0026	0.013161

	regionalization	GO:0003002	304	17	0.00297	0.01488
	organic hydroxy compound biosynthetic process	GO:1901617	231	14	0.00334	0.016498
	positive regulation of reactive oxygen species metabolic process	GO:2000379	24	4	0.00336	0.016498
	phospholipid metabolic process	GO:0006644	160	11	0.00349	0.016848
	**hormone metabolic process**	**GO:0042445**	**362**	**19**	**0.0035**	**0.016848**
	amino acid export across plasma membrane	GO:0032973	4	2	0.00405	0.019306
	cellular response to metal ion	GO:0071248	80	7	0.00517	0.024408
	regulation of defense response to virus	GO:0050688	14	3	0.00538	0.025158
**KEGG term**	03019 Messenger RNA biogenesis	-	750	73	1.11E-16	8.10E-15
	**00073 Cutin, suberine and wax biosynthesis**	**-**	**148**	**30**	**3.33E-16**	**1.22E-14**
	**00199 Cytochrome P450**	**-**	**350**	**59**	**4.44E-16**	**1.08E-14**
	03013 Nucleocytoplasmic transport	-	267	71	4.44E-16	1.08E-14
	03010 Ribosome	-	406	58	5.55E-16	8.10E-15
	00908 Zeatin biosynthesis	-	83	29	5.55E-16	8.10E-15
	99981 Carbohydrate metabolism	-	26	14	7.77E-16	8.10E-15
	03011 Ribosome	-	432	58	1.11E-15	1.01E-14
	B 09122 Translation	-	985	72	1.22E-15	9.91E-15
	B 09181 Protein families: metabolism	-	3033	142	4.63E-14	3.38E-13
	03012 Translation factors	-	297	31	4.47E-11	2.72E-10
	04626 Plant-pathogen interaction	-	355	33	2.28E-10	1.28E-09
	B 09109 Metabolism of terpenoids and polyketides	-	443	36	1.39E-09	6.77E-09
	99980 Enzymes with EC numbers	-	709	47	3.32E-09	1.51E-08
	A09150 Organismal Systems	-	421	33	1.69E-08	7.26E-08
	**B 09159 Environmental adaptation**	**-**	**421**	**33**	**1.69E-08**	**7.26E-08**
	01003 Glycosyltransferases	-	404	31	7.59E-08	2.91E-07
	B 09103 Lipid metabolism	-	844	45	3.62E-06	1.32E-05
	00592 alpha-Linolenic acid metabolism	-	140	15	3.64E-06	1.26E-05
	01002 Peptidases and inhibitors	-	604	35	8.11E-06	2.69E-05
	00260 Glycine, serine and threonine metabolism	-	181	16	2.06E-05	6.54E-05
	03029 Mitochondrial biogenesis	-	724	36	1.48E-04	4.51E-04
	00240 Pyrimidine metabolism	-	98	8	0.003989	0.011648
	**00906 Carotenoid biosynthesis**	**-**	**75**	**6**	**0.013255**	**0.037216**

Note: GO and KEGG terms in bold may be related to the adaptation of *C. gigantea* to high altitude.

Supplementary Table S11 GO and KEGG enrichment of *Cupressus gigantea* specific gene families

	Term	ID	Annotated	Significant	*P* value	Adjust *P* value
GO term	regulation of adaxial/abaxial pattern formation	GO:2000011	16	8	4.80E-08	9.31E-05
	negative regulation of cytokinin-activated signaling pathway	GO:0080037	28	8	7.60E-06	0.00388
	response to chemical	GO:0042221	3138	162	8.60E-06	0.00388
	**regulation of phenylpropanoid metabolic process**	**GO:2000762**	**67**	**12**	**8.70E-06**	**0.00388**
	**response to hormone**	**GO:0009725**	**1725**	**100**	**1.20E-05**	**0.00388**
	isoleucyl-tRNA aminoacylation	GO:0006428	15	6	1.20E-05	0.00388
	**response to endogenous stimulus**	**GO:0009719**	**1761**	**101**	**1.70E-05**	**0.00471**
	response to organic substance	GO:0010033	2162	118	2.60E-05	0.0063
	defense response to bacterium	GO:0042742	631	45	5.20E-05	0.00847
	sulfur compound biosynthetic process	GO:0044272	106	14	5.80E-05	0.00847
	glycosyl compound biosynthetic process	GO:1901659	81	12	6.30E-05	0.00847
	olefin metabolic process	GO:1900673	47	9	6.80E-05	0.00847
	regulation of cell communication	GO:0010646	415	33	7.20E-05	0.00847
	regulation of signaling	GO:0023051	415	33	7.20E-05	0.00847
	tRNA aminoacylation for protein translation	GO:0006418	20	6	8.10E-05	0.00847
	amino acid activation	GO:0043038	20	6	8.10E-05	0.00847
	regulation of signal transduction	GO:0009966	400	32	8.30E-05	0.00847
	**response to anoxia**	**GO:0034059**	**22**	**6**	**0.00015**	**0.01454**
	response to bacterium	GO:0009617	717	47	0.00026	0.0238
	negative regulation of signal transduction	GO:0009968	152	16	0.00027	0.0238
	negative regulation of cell communication	GO:0010648	154	16	0.00032	0.02439
	regulation of secondary metabolic process	GO:0043455	96	12	0.00033	0.02439
	fungal-type cell wall organization	GO:0031505	10	4	0.00039	0.02439
	sepal giant cell differentiation	GO:0090392	10	4	0.00039	0.02439
	plant epidermal cell fate specification	GO:0090628	10	4	0.00039	0.02439
	self proteolysis	GO:0097264	10	4	0.00039	0.02439
	negative regulation of response to stimulus	GO:0048585	327	26	0.00042	0.02545
	organonitrogen compound biosynthetic process	GO:1901566	633	42	0.00044	0.02585
	**hormone transport**	**GO:0009914**	**114**	**13**	**0.00047**	**0.02639**
	response to auxin	GO:0009733	385	29	0.00049	0.02639
	**response to decreased oxygen levels**	**GO:0036293**	**130**	**14**	**0.00051**	**0.02653**
	amide biosynthetic process	GO:0043604	177	17	0.00052	0.02653
	cellular response to organic substance	GO:0071310	949	57	0.00055	0.02715
	**response to oxygen levels**	**GO:0070482**	**131**	**14**	**0.00056**	**0.02715**
	mRNA modification	GO:0016556	88	11	0.00058	0.02743
	shikimate metabolic process	GO:0019632	11	4	0.0006	0.0277
	auxin transport	GO:0060918	103	12	0.00063	0.02841
	secondary metabolic process	GO:0019748	392	29	0.00065	0.02864
	**response to hydrogen peroxide**	**GO:0042542**	**151**	**15**	**0.00078**	**0.03007**
	**telomere capping**	**GO:0016233**	**12**	**4**	**0.00087**	**0.03007**
	nucleotide salvage	GO:0043173	12	4	0.00087	0.03007
	ethylene metabolic process	GO:0009692	30	6	0.00089	0.03007

	cellular alkene metabolic process	GO:0043449	30	6	0.00089	0.03007
	olefin biosynthetic process	GO:1900674	30	6	0.00089	0.03007
	cellular response to cytokinin stimulus	GO:0071368	66	9	0.00096	0.03007
	UMP biosynthetic process	GO:0006222	6	3	0.00107	0.03007
	biotin metabolic process	GO:0006768	6	3	0.00107	0.03007
	pyrimidine nucleoside monophosphate metabolic process	GO:0009129	6	3	0.00107	0.03007
	pyrimidine ribonucleoside monophosphate metabolic process	GO:0009173	6	3	0.00107	0.03007
	pyrimidine ribonucleotide salvage	GO:0010138	6	3	0.00107	0.03007
	pyrimidine nucleotide salvage	GO:0032262	6	3	0.00107	0.03007
	**cellular response to hormone stimulus**	**GO:0032870**	**726**	**45**	**0.00117**	**0.03154**
	response to karrikin	GO:0080167	207	18	0.00117	0.03154
	primary miRNA processing	GO:0031053	13	4	0.00122	0.03154
	regulation of morphogenesis of a branching structure	GO:0060688	13	4	0.00122	0.03154
	regulation of secondary shoot formation	GO:2000032	13	4	0.00122	0.03154
	triglyceride metabolic process	GO:0006641	43	7	0.00122	0.03154
	**hormone-mediated signaling pathway**	**GO:0009755**	**571**	**37**	**0.00149**	**0.03615**
	epidermal cell differentiation	GO:0009913	33	6	0.00151	0.03615
	chloroplast fission	GO:0010020	33	6	0.00151	0.03615
	**activation of MAPK activity**	**GO:0000187**	**23**	**5**	**0.00163**	**0.03854**
	fatty acid derivative biosynthetic process	GO:1901570	58	8	0.0017	0.03971
	epidermis development	GO:0008544	34	6	0.00177	0.0401
	cellular response to sucrose starvation	GO:0043617	7	3	0.00182	0.0401
	positive regulation of defense response to bacterium	GO:1902479	7	3	0.00182	0.0401
	cellular aromatic compound metabolic process	GO:0006725	2631	128	0.00188	0.04093
	**cellular response to endogenous stimulus**	**GO:0071495**	**767**	**46**	**0.00201**	**0.04236**
	regulation of endosperm development	GO:2000014	15	4	0.00218	0.0447
	**response to reactive oxygen species**	**GO:0000302**	**291**	**22**	**0.00219**	**0.0447**
KEGG term	**00944 Flavone and flavonol biosynthesis**	**-**	**13**	**8**	**3.19E-09**	**4.78E-07**
	B 09181 Protein families: metabolism	-	3033	158	4.44E-07	3.33E-05
	00780 Biotin metabolism	-	34	9	2.42E-06	1.21E-04
	**00199 Cytochrome P450**	**-**	**350**	**31**	**4.65E-06**	**1.74E-04**
	01007 Amino acid related enzymes	-	169	19	1.27E-05	3.81E-04
	01001 Protein kinases	-	743	49	3.99E-05	9.97E-04

Note: GO and KEGG terms in bold may be related to the adaptation of *C. gigantea* to high altitude.

Supplementary Table S12 Information on populations and individuals of *Cupressus gigantea* and *C. duclouxiana* used in re-sequencing

Species	Population ID	Sample ID	Location	Longitude (°E)	Latitude (°N)	Elevation (m)
C. gigantea	BY	MaoKS-QTP-2018-32	Bayi, Tibet, China	29.6291	94.3915	3150
		WLZ-1-4	Bayi, Tibet, China	29.6291	94.3915	3150
		WLZ-1-6	Bayi, Tibet, China	29.6291	94.3915	3150
		WLZ-1-7	Bayi, Tibet, China	29.6291	94.3915	3150
		WLZ-1-9	Bayi, Tibet, China	29.6291	94.3915	3150
		WLZ-1-15	Bayi, Tibet, China	29.6291	94.3915	3150
		WLZ-1-17	Bayi, Tibet, China	29.6291	94.3915	3150
	ML1	WLZ-11-1	Milin, Tibet, China	29.3601	94.3988	2875
		WLZ-11-4	Milin, Tibet, China	29.3601	94.3988	2875
		WLZ-11-5	Milin, Tibet, China	29.3601	94.3988	2875
		WLZ-11-7	Milin, Tibet, China	29.3601	94.3988	2875
	ML2	WLZ-2-1	Milin, Tibet, China	29.1258	93.8560	2994
		WLZ-2-2	Milin, Tibet, China	29.1258	93.8560	2994
		WLZ-2-4	Milin, Tibet, China	29.1258	93.8560	2994
	ML3	MaoKS-QTP-2018-29-1	Milin, Tibet, China	29.1603	93.6652	3133
		MaoKS-QTP-2018-29-5	Milin, Tibet, China	29.1603	93.6652	3133
	ML4	WLZ-10-1	Milin, Tibet, China	29.1494	93.4632	2981
		WLZ-10-2	Milin, Tibet, China	29.1494	93.4632	2981
		WLZ-10-3	Milin, Tibet, China	29.1494	93.4632	2981
	LX1	WLZ-8-1	Langxian Tibet, China	29.0071	93.3152	3021
		WLZ-8-4	Langxian Tibet, China	29.0071	93.3152	3021
		WLZ-8-7	Langxian Tibet, China	29.0071	93.3152	3021
		MaoKS-QTP-2018-23	Langxian Tibet, China	29.0071	93.3152	3021
	LX2	WLZ-5-4	Langxian Tibet, China	29.1335	93.1426	3067
		WLZ-5-5	Langxian Tibet, China	29.1335	93.1426	3067
		WLZ-5-7	Langxian Tibet, China	29.1335	93.1426	3067
	LX3	WLZ-4-2	Langxian Tibet, China	29.0646	93.0756	3045
		WLZ-4-4	Langxian Tibet, China	29.0646	93.0756	3045
		WLZ-4-9	Langxian Tibet, China	29.0646	93.0756	3045
		WLZ-4-12	Langxian Tibet, China	29.0646	93.0756	3045
	LX4	WLZ-3-1	Langxian Tibet, China	29.0692	92.9326	3110
		WLZ-3-2	Langxian Tibet, China	29.0692	92.9326	3110
C. duclouxiana	DQ1	DQ-1-2	Deqing, Yunnan, China	28.2210	98.8561	1966
		DQ-1-5	Deqing, Yunnan, China	28.2210	98.8561	1966
		DQ-1-6	Deqing, Yunnan, China	28.2210	98.8561	1966
	DQ2	DQ-2-1	Deqing, Yunnan, China	28.3756	98.8851	2540
		DQ-2-6	Deqing, Yunnan, China	28.3756	98.8851	2540
		DQ-2-10	Deqing, Yunnan, China	28.3756	98.8851	2540
	DQ3	DQ-3-1	Deqing, Yunnan, China	28.4893	98.0824	2754
		DQ-3-5	Deqing, Yunnan, China	28.4893	98.0824	2754
		DQ-3-8	Deqing, Yunnan, China	28.4893	98.0824	2754

DQ4	DQ-4-1	Deqing, Yunnan, China	28.2644	99.2315	2722
	DQ-4-2	Deqing, Yunnan, China	28.2644	99.2315	2722
	DQ-4-3	Deqing, Yunnan, China	28.2644	99.2315	2722
KG	KG-1	Shangri-La, Yunnan, China	28.0209	99.4328	2870
	KG-3	Shangri-La, Yunnan, China	28.0209	99.4328	2870
	KG-4	Shangri-La, Yunnan, China	28.0209	99.4328	2870
	KG-6	Shangri-La, Yunnan, China	28.0209	99.4328	2870
DSX	DSX-1-3	Shangri-La, Yunnan, China	28.6963	99.7645	3155
	DSX-1-4	Shangri-La, Yunnan, China	28.6963	99.7645	3155
	DSX-1-6	Shangri-La, Yunnan, China	28.6963	99.7645	3155
	DSX-1-8	Shangri-La, Yunnan, China	28.6963	99.7645	3155
DC	M65-1	Dechang, Sichuan, China	27.5369	102.1944	2256
	M64-5	Dechang, Sichuan, China	27.5369	102.1944	2256
	M64-1	Dechang, Sichuan, China	27.5369	102.1944	2256
	M66-1	Dechang, Sichuan, China	27.5369	102.1944	2256
HZ	HZ-1	Qujing, Yunnan, China	26.4269	103.3416	2156
	HZ-5	Qujing, Yunnan, China	26.4269	103.3416	2156
DSC	DSC-4	Kunming, Yunnan, China	25.2468	102.7382	2159
	DSC-1	Kunming, Yunnan, China	25.2468	102.7382	2159
	XLHS-8	Kunming, Yunnan, China	25.2468	102.7382	2159
ML	ML-1-4	Mile, Yunan, China	24.4314	103.3948	1659
	ML-1-8	Mile, Yunan, China	24.4314	103.3948	1659
	ML-1-10	Mile, Yunan, China	24.4314	103.3948	1659
MZ1	MZ-1-4	Mengzi, Yunnan, China	23.3151	103.6590	1605
	MZ-1-8	Mengzi, Yunnan, China	23.3151	103.6590	1605
MZ2	MZ-2-2	Mengzi, Yunnan, China	23.2434	103.3712	1596
	MZ-3-1	Mengzi, Yunnan, China	23.2434	103.3712	1596
	MZ-3-4	Mengzi, Yunnan, China	23.2434	103.3712	1596
WS	WS-1-5	Wenshan, Yunan, China	23.4346	104.6972	1648
	WS-1-7	Wenshan, Yunan, China	23.4346	104.6972	1648
	XC-1-3	Wenshan, Yunan, China	23.4346	104.6972	1648
PP	PP-1-1	Dali, Yunnan, China	25.5844	100.0517	1860
	PP-1-6	Dali, Yunnan, China	25.5844	100.0517	1860
	BD-1	Dali, Yunnan, China	25.5844	100.0517	1860
LJ	LJ-3-2	Lijiang, Yunnan, China	27.0004	100.2066	2405
	JC-2-3	Lijiang, Yunnan, China	27.0004	100.2066	2405
	JC-2-8	Lijiang, Yunnan, China	27.0004	100.2066	2405
HTX	HTX-1	Shangri-La, Yunnan, China	27.2684	100.1694	1969
	HTX-8	Shangri-La, Yunnan, China	27.2684	100.1694	1969
KS	KS-1	Daocheng, Sichuan, China	28.3974	100.2432	2833
	KS-4	Daocheng, Sichuan, China	28.3974	100.2432	2833
	KS-7	Daocheng, Sichuan, China	28.3974	100.2432	2833

Supplementary Table S13 Reads and mapping statistics results

Species	Sample ID	Raw data (Gb)	Clean data (Gb)	Q20 (%)	GC content (%)	Covered sites	Genome coverage (%)	Effective depth (X)
C. gigantea	MaoKS-QTP-2018-32	150.55	147.74	97.26	35.32	9353760000	85.6609	12.8509
	WLZ-1-4	160.43	158.15	96.81	36.18	9271080000	84.9038	13.2109
	WLZ-1-6	178.85	175.61	96.44	35.42	9263328516	84.8329	12.1459
	WLZ-1-7	317.33	308.62	95.91	35.17	9441851421	86.4678	25.791
	WLZ-1-9	149.65	146.94	96.33	35.40	9229663697	84.5246	12.4896
	WLZ-1-15	191.25	186.14	95.46	35.45	9405920000	86.1385	15.9859
	WLZ-1-17	179.55	176.28	96.08	35.36	9292418064	85.0993	15.0312
	WLZ-11-1	169.54	167.05	96.76	36.03	9303086415	85.197	14.2215
	WLZ-11-4	159.99	157.28	96.67	35.23	9270120000	84.8949	13.6092
	WLZ-11-5	158.74	156.16	96.22	35.23	9227086695	84.501	13.317
	WLZ-11-7	172.11	168.98	96.28	35.40	9247811906	84.6908	14.2383
	WLZ-2-1	164.14	161.65	96.41	36.26	9340630000	85.5407	13.462
	WLZ-2-2	181.47	178.35	96.15	35.34	9362848839	85.7443	15.1877
	WLZ-2-4	172.00	168.88	96.07	35.53	9300367460	85.1721	14.2091
	MaoKS-QTP-2018-29-1	178.53	176.74	96.88	35.43	9396140000	86.049	15.4994
	MaoKS-QTP-2018-29-5	161.36	158.67	97.42	35.46	9302710000	85.1934	13.6958
	WLZ-10-1	153.16	151.26	96.71	36.01	9323030000	85.3795	12.8314
	WLZ-10-2	192.19	189.59	95.40	35.61	9329930000	85.4426	16.0357
	WLZ-10-3	161.34	158.86	96.13	35.61	9347220000	85.601	13.4181
	WLZ-8-1	163.51	160.82	96.26	35.56	9261100000	84.8123	13.5767
	WLZ-8-4	185.55	182.46	96.53	35.72	9303120000	85.1972	15.14
	WLZ-8-7	158.69	155.73	96.01	35.52	9291660000	85.0922	13.0158
	MaoKS-QTP-2018-23	135.59	131.36	97.14	35.44	9419290000	86.261	14.9034
	WLZ-5-4	162.21	159.02	96.00	35.50	9302510000	85.1915	13.3007
	WLZ-5-5	190.40	187.12	96.06	35.53	9291010000	85.0862	15.6512
	WLZ-5-7	169.46	166.57	96.01	35.57	9287370000	85.0529	13.9688
	WLZ-4-2	160.76	157.81	96.02	35.64	9243150000	84.6479	13.1944
	WLZ-4-4	164.38	161.54	96.17	35.67	9292450000	85.0995	13.4626
	WLZ-4-9	166.40	163.29	96.18	35.52	9256230000	84.7677	13.7139
	WLZ-4-12	248.62	244.84	96.59	35.47	9330980000	85.4523	20.5608
	WLZ-3-1	149.70	147.20	96.40	35.37	9195343709	84.2103	12.4212
	WLZ-3-2	184.04	180.64	96.25	35.64	9252790000	84.7362	15.2096
C. duclouxiana	DQ-1-2	160.62	157.10	95.43	35.18	8847390000	81.0236	13.2366
	DQ-1-5	173.82	170.69	96.07	35.37	8721764994	79.8733	14.2312
	DQ-1-6	148.92	146.19	96.19	35.49	8603244741	78.7879	12.1459
	DQ-2-1	198.42	195.00	96.18	35.37	8753737290	80.1661	16.2135
	DQ-2-6	178.51	175.14	96.27	35.43	8683448468	79.5224	14.4565
	DQ-2-10	152.79	150.00	96.23	35.43	8619613071	78.9378	12.3141
	DQ-3-1	142.97	140.79	96.35	35.71	8586068367	78.6306	11.7803
	DQ-3-5	158.13	155.68	96.21	35.25	8599106250	78.75	12.9327
	DQ-3-8	220.80	216.99	95.81	35.34	8819723828	80.7704	17.8035
	DQ-4-1	151.16	148.61	96.44	35.28	8613585507	78.8826	12.2515
	DQ-4-2	166.23	163.77	96.43	35.35	8660364645	79.311	13.4475

	DQ-4-3	151.54	149.09	96.30	35.32	8640490000	79.1288	12.1647
	KG-1	154.74	152.72	96.76	35.47	8732640000	79.9727	12.667
	KG-3	157.63	155.25	96.06	35.66	8704580000	79.7158	12.907
	KG-4	153.10	150.75	96.40	35.55	8648660000	79.2037	12.5013
	KG-6	154.91	152.53	96.53	35.37	8700030000	79.6741	12.4638
	DSX-1-3	150.67	150.37	91.65	36.24	8932760000	81.8054	12.2358
	DSX-1-4	151.72	151.28	92.50	36.20	8909700000	81.5942	12.3322
	DSX-1-6	163.32	163.13	92.75	36.15	8915820000	81.6503	13.1255
	DSX-1-8	160.81	160.60	92.71	36.06	8910530000	81.6018	13.1839
	M65-1	165.33	162.41	96.49	35.96	8580100000	78.5758	13.2059
	M64-5	179.66	178.29	97.54	35.63	8690540000	79.5872	15.0606
	M64-1	199.16	195.74	96.44	35.29	8809920000	80.6805	16.0626
	M66-1	156.03	152.93	96.21	35.47	8543850000	78.2438	12.6529
	HZ-1	195.10	194.88	92.41	36.36	8854690000	81.0904	15.3912
	HZ-5	172.30	172.08	91.93	36.31	8828710000	80.8525	13.659
	DSC-4	171.02	170.85	94.68	35.64	8785010000	80.4523	13.8574
	DSC-1	189.38	189.09	91.90	36.36	8743710000	80.0741	15.1127
	XLHS-8	164.31	164.07	92.77	36.16	8783580000	80.4392	13.3037
	ML-1-4	197.62	197.32	92.80	36.15	8860710000	81.1456	15.8408
	ML-1-8	142.20	141.99	92.44	36.18	8725010000	79.9029	14.6297
	ML-1-10	152.44	152.14	92.69	36.13	8723260000	79.8868	12.397
	MZ-1-4	151.26	151.04	93.52	36.12	8722560000	79.8804	12.2901
	MZ-1-8	196.30	196.02	92.65	36.32	8815080000	80.7277	15.571
	MZ-2-2	151.45	151.23	91.81	36.30	8732060000	79.9674	11.8557
	MZ-3-1	168.07	165.68	96.52	36.25	8587370000	78.6424	13.2685
	MZ-3-4	186.62	186.44	94.43	35.95	8766820000	80.2858	14.8571
	WS-1-5	162.50	162.33	93.85	35.96	8804000000	80.6262	15.7785
	WS-1-7	195.63	195.44	95.19	35.70	8804000000	80.6262	15.7785
	XC-1-3	166.34	166.19	94.48	35.88	8741750000	80.0561	13.4625
	PP-1-1	184.98	184.80	94.18	35.77	8815680000	80.7332	15.1515
	PP-1-6	181.22	181.05	94.58	35.66	8821980000	80.7909	14.9398
	BD-1	211.92	211.71	94.66	35.80	8889090000	81.4054	17.061
	LJ-3-2	148.79	148.65	95.06	35.55	8759780000	80.2213	12.1057
	JC-2-3	151.22	150.85	94.91	35.85	8631070000	79.0425	12.2968
	JC-2-8	133.89	133.76	94.81	35.89	8857920000	81.1201	16.9147
	HTX-1	152.96	152.42	95.34	35.48	8702320000	79.6951	12.5347
	HTX-8	120.72	120.62	95.21	35.50	8847410000	81.0238	17.4217
	KS-1	162.97	162.79	93.60	35.98	8906240000	81.5626	13.1416
	KS-4	150.61	150.31	94.64	35.72	8847650000	81.026	12.211
	KS-7	182.80	182.61	93.81	35.91	8972440000	82.1688	14.9168
Juniperus microsperma	MaoKS-QTP-2018-08-3	176.09	173.68	96.48	35.26	6267020000	57.3928	11.9841
	LXT-05-8	164.29	161.59	96.49	35.53	8157910000	74.7094	13.0044
C. chengiana	LXT-08-3	160.52	158.89	92.49	36.14	8524010000	78.0621	17.2264
	LXT-10-5	161.70	161.55	93.69	35.42	8437500000	77.2699	12.9917

LXT-18-11	198.37	198.18	93.40	35.53	8568690000	78.4713	15.9247
LXT-21-9	151.55	150.95	92.52	35.78	8413870000	77.0534	12.1468

Supplementary methods

Estimation of genome size based on 25-mer analysis
The genome size was estimated by 25-*k*-mer frequency analysis based on short reads. Briefly, SOAPnuke (ver. 1.6.5) was used to remove low-quality and redundant DNBSEQ reads [1]. All clean reads were selected to generate *k*-mer frequency distribution using Jellyfish (ver. 2.1.4) [2], and the genome size was calculated based on the following formula: $G = N_{kmer}/C_{kmer}$ (N_{kmer} and C_{kmer} are the total numbers and the average coverage depth of *k*-mers, respectively). GenomeScope (ver. 2.0) was then used to estimate the overall characteristics of the genome, including genome size, heterozygous ratio and repeat sequence ratio [3].

LTR insertion time estimation
To explore the dynamics of long terminal repeat retrotransposons (LTR-RTs), full_length of LTR_RTs was initially searched by LTR_FINDER (ver. 1.02) [4] and LTRharvest [5]. LTR_retriever (ver. 2.9.0) was then used to integrate these results and remove non-LTR_RTs or those with large amounts of tandem repeats or gaps [6]. Finally, the time of insertion (*T*) of intact LTR-RTs was estimated by LTR_retriever using the formula $T = K/2\mu$ where K is the divergence rate and μ is the nucleotide substitution rate. The assumed substitution rate for *Oryza sativa* was 1.31×10^{-8} and that for *Picea abies*, *Gnetum montanum* and *C. gigantea* was 2.2×10^{-9} [7, 8]. To construct Ty1/*Copia* and Ty3/*Gypsy* phylogenetic trees, these intact LTR_RTs were translated into amino acids in three-frames form. Translated sequences were then mapped against the Ty1_*Copia* (PF07727) and Ty3_*Gypsy* (PF00078) domains in the Pfam database (http://pfam-legacy.xfam.org/) using HMMER (http://hmmer.org), with E-values ≤ 1e-5. The resultant amino acid sequences were aligned using MAFFT (ver. 7.429) with default parameters [9]. Heuristic neighbor-joining (NJ) trees for two superfamilies were built based on multiple sequence alignment using in FastTree [10].

Gene prediction
For the *ab initio*-based gene annotation, we used AUGUSTUS (ver. 3.3.1) [11], GlimmerHMM (ver. 3.0.4) [12], SNAP [13], Genscan (ver. 1.0) [14], FgeneSH (ver. 2.0) [15], and GeneID (ver. 1.4.4) [16] with default parameters. The unigenes assembled from the RNA-seq data were used as the training set. Protein sequences of *Picea abies*, *Picea glauca*, *Picea sitchensis*, *Pinus taeda*, and *Sequoiadendron giganteum* were downloaded from National Center for Biotechnology Information (NCBI, https://www.ncbi.nlm.nih.gov/genome); Protein sequences of *Pinus pinaster*, *Pinus sylvestris*, *Pseudostsuga menziesii* and *Taxus baccata* were download from PLAZA (https://bioinformatics.psb.ugent.be/plaza/versions/gymno-plaza/). Protein sequences of *Pinus tabuliformis* were downloaded from Figshare (https://figshare.com/articles/dataset/P_tabuliformis_V1_0_Protein/19060865). These species were chosen for homology annotation using Exonerate (ver. 2.2.0) [17] and GeneWise (ver. 2.4.1) [18]. In the RNA-seq-based prediction, quality-controlled reads from all RNA libraries were mapped to the *C. gigantea* genome using HISAT2 [19]. Finally, a consensus gene set was generated by integrating the results from three approaches by EVidenceModeler (EVM; ver. 1.1.1) [20]. In addition, we removed genes that had too long repetitive sequences (>50%) or were too short (<=50 amino acids) to obtain a high-confidence annotated gene set.

Genome evolution
We applied two methods to detect whole-genome duplication (WGD) events. In the first method, synonymous substitution rates per gene (Ks) between collinear genes were calculated using the *KaKs*_Calculator (ver. 2.0) with default parameters [21], and the distribution of Ks values was used to determine events of WGD. In the second method, the four-fold synonymous third-codon transversion rates (4DTv) of syntenic blocks were estimated and used to detect WGD. Furthermore, Circos (ver. 0.69.8) was used to illustrate the positional relationships among synteny blocks within the *C. gigantea* genome[22].

We downloaded the protein sequences of *Arabidopsis thaliana*, *Vitis vinifera*, *Oryza sativa*, *Amborella trichopoda*, *Selaginella moellendorffii* and *Physcomitrium patens* from Phytozome (https://phytozome-next.jgi.doe.gov/); protein sequences of *Ginkgo biloba* from Figshare (https://figshare.com/articles/dataset/annotation_of_Ginkgo_biloba/14759223). Orthologous gene

family in *C. gigantea* and these nine species were identified by using OrthoFinder (ver. 2.5.2) [23]. In total, 319 high confidence single-copy orthologues genes were extracted, again aligned with MAFFT [9], and analyzed phylogenetically using RaxML (ver. 8.2.12) with 1000 bootstraps [24]. Divergence time was estimated using MCMCtree in PAML (ver. 4.9j) software package [25], using two calibrations, namely the divergence of *A. thaliana* and *V. vinifera* (115-105 million years ago [Mya]) and the divergence of angiosperms and gymnosperms (330-289 Mya) obtained from the TimeTree database (http://www.timetree.org/). After a burn-in of 1,000,000 iterations, MCMC chains were run for 50,000 generations, with a sampling frequency of 1,000. To explore the expansion and contraction of gene families in *C. gigantea*, CAFE (ver. 4.2.1) was used with the *P*-value set to 0.01, and auto searching for the λ value (lambda, representing the average gene birth and death rate) [26]. Subsequently, GO enrichment analyses were performed using *R* package topGO to identify significantly enriched terms [27]. Fisher's exact test was applied to calculate the statistical significance of enrichment. In addition, KEGG enrichment analyses were performed using TBtools [28]. The resulting *P*-values were corrected using the method of Benjamini & Hochberg (1995) [29].

Theoretical predictions of the derived count and population fitness

The overall number of derived haploid deleterious through generations (t) was calculated according to Eq. 4 in Kleinman-Ruiz et al. (2022) [30]:

$$L_t = \frac{gt}{ft} \sum q_0(1\text{-}f_t) + \sum q_1[1\text{-}\frac{gt}{ft}(1\text{-}f_t)] + t(\lambda 2 N_{new} U)$$

$\sum q_0$ and $\sum q_1$ are approximated numbers of derived count in ancestral population (N_{anc}) and new population (N_{new}) approaches a mutation-selection-drift (MSD) balance, respectively. They are calculated by Eq. 2, 3 in Kleinman-Ruiz et al. (2022) [30]:

$$\sum q_0 = \frac{\lambda}{hs + K_0 s(1-2h)}$$

$$\sum q_1 = \frac{\lambda}{hs + K_1 s(1-2h)}$$

where λ is deleterious mutation rate per gamete and generation [31], s is the selection coefficients, h is the dominance coefficients and $K = \frac{\sum q^2}{\sum q}$ (Eq. 9 in García-Dorado, 2007 [31]) is the proportion of deleterious copies suffering selection under homozygous condition. ft is the standard Wright inbreeding coefficient and g_t is the purged inbreeding coefficient (Eq. 2 in García-Dorado 2012 [32]). U is the new fixation probability for a new mutation. Using $\sum q$, ft and g_t the equation still fails to describe the change in deleterious burden in the transition from the ancestral to the new balance because the continuous effects of genetic drift are little described. At the new MSD balance, a population with effective population size N_{new} generates 2 λ N_{new} for each generation (t). Take U as the fixation probability for a new mutation (Eq. 3 in Kimura 1962 [33]), $t(\lambda 2 N_{new} U)$ is to represent the deleterious fixation at the new MSD balance thus approximate the continuous increase in genetic load caused mainly by genetic drift.

Predictions are obtained separately for each period of constant N_e, with the corresponding parameters (e.g. ft and g_t) recalculated. Because of the frequent changes in N_e estimated by SMC++, we simplified the N_e used for the predictions by retaining only a few significant N_e declines and expansions. The prediction is not an empirical calculation due to the introduction of approximations, including the absence of LD, but it provides illustrative predictions.

The prediction on the fitness (w) was computed according to Eq. 13 in García-Dorado (2012) [32]:

$$w_t = w_0 - (L_0 - L_t)g_t - (t - 2Nf_t)D_m$$

where D_m is the fitness decline due to deleterious fixation. We assumed that the ancestral N_e was larger enough (here, N_e = 1,000,000) to make the rate of fitness decline negligible during MSD balance, thus w_0 = 1. To explore the effect of different N_e on the rate of decline in fitness, we setup different N_{new} (N_e = 6558, 3500, 2500, and 1000) to run the prediction.

Supplementary references

1. Chen YX, Chen YS, Shi CM et al. SOAPnuke: a MapReduce acceleration-supported software for integrated quality control and preprocessing of high-throughput sequencing

data. *GigaScience.* 2018; **7:** 1-6.
2. Marcais G, Kingsford C. A fast, lock-free approach for efficient parallel counting of occurrences of k-mers. *Bioinformatics.* 2011; **27:** 764-770.
3. Vurture GW, Sedlazeck FJ, Nattestad M et al. GenomeScope: fast reference-free genome profiling from short reads. *Bioinformatics.* 2017; **33:** 2202-2204.
4. Xu Z, Wang H. LTR_FINDER: an efficient tool for the prediction of full-length LTR retrotransposons. *Nucleic Acids Res.* 2007; **35:** W265-W268.
5. Ellinghaus D, Kurtz S, Willhoeft U. LTRharvest, an efficient and flexible software for de novo detection of LTR retrotransposons. *BMC Bioinform.* 2018; **9:** 18.
6. Ou SJ, Jiang N. LTR_retriever: a highly accurate and sensitive program for identification of long terminal repeat retrotransposons. *Plant Physiol.* 2018; **176:** 1410-1422.
7. Ma JX, Bennetzen JL. Rapid recent growth and divergence of rice nuclear genomes. *Proc Natl Acad Sci USA* 2004; **101:** 12404-12410.
8. Nystedt B, Street NR, Wetterbom A et al. The Norway spruce genome sequence and conifer genome evolution. *Nature.* 2013; **497:** 579-584.
9. Katoh K, Standley DM. MAFFT multiple sequence alignment software version 7: improvements in performance and usability. *Mol Biol Evol.* 2013; **30:** 772-780.
10. Price MN, Dehal PS, Arkin AP. FastTree 2--approximately maximum-likelihood trees for large alignments. *PloS One.* 2010; **5:** e9490.
11. Stanke M, Steinkamp R, Waack S et al. AUGUSTUS: a web server for gene finding in eukaryotes. *Nucleic Acids Res.* 2004; **32:** W309-W312.
12. Majoros WH, Pertea M, Salzberg SL. TigrScan and GlimmerHMM: two open source ab initio eukaryotic gene-finders. *Bioinformatics.* 2004; **20:** 2878-2879.
13. Johnson AD, Handsaker RE, Pulit SL et al. SNAP: a web-based tool for identification and annotation of proxy SNPs using HapMap. *Bioinformatics.* 2008; **24:** 2938-2939.
14. Burge C, Karlin S. Prediction of complete gene structures in human genomic DNA. *J Mol Biol.* 1997; **268:** 78-94.
15. Solovyev V, Kosarev P, Seledsov I et al. Automatic annotation of eukaryotic genes, pseudogenes and promoters. *Genome Biol.* 2006; **7:** S10.
16. Blanco E, Parra G, Guigó R. Using geneid to identify genes. *Curr. Protoc. Bioinformatics.* 2007; **Chapter 4:** Unit 4.3.
17. Slater GSC, Birney E. Automated generation of heuristics for biological sequence comparison. *BMC Bioinform.* 2005; **6:** 31.
18. Birney E, Clamp M, Durbin R. GeneWise and Genomewise. *Genome Res.* 2004; **14:** 988-995.
19. Kim D, Langmead B, Salzberg SL. HISAT: a fast spliced aligner with low memory requirements. *Nat Methods.* 2015; **12:** 357-360.
20. Haas BJ, Salzberg SL, Zhu W et al. Automated eukaryotic gene structure annotation using EVidenceModeler and the program to assemble spliced alignments. *Genome Biol.* 2008; **9:** R7.
21. Wang DP, Zhang YB, Zhang Z et al. KaKs_Calculator 2.0: a toolkit incorporating gamma-series methods and sliding window strategies. *Genom Proteom Bioinf.* 2010; **8:** 77-80.
22. Krzywinski M, Schein J, Birol I et al. Circos: an information aesthetic for comparative genomics. *Genome Res.* 2009; **19:** 1639-1645.
23. Emms DM, Kelly S. OrthoFinder: phylogenetic orthology inference for comparative genomics. *Genome Biol.* 2019; **20:** 238.
24. Stamatakis A. RAxML version 8: a tool for phylogenetic analysis and post-analysis of large phylogenies. *Bioinformatics.* 2014; **30:** 1312-1313.
25. Yang ZH. PAML 4: Phylogenetic analysis by maximum likelihood. *Mol Biol Evol.* 2007; **24:** 1586-1591.
26. De Bie T, Cristianini N, Demuth JP et al. CAFE: a computational tool for the study of gene family evolution. *Bioinformatics.* 2006; **22:** 1269-127.
27. Alexa A, Rahnenführer J. Gene set enrichment analysis with topGO. *Bioconductor.* 2009; **27**.
28. Chen CJ, Chen H, Zhang Y et al. TBtools: an integrative toolkit developed for interactive analyses of big biological data. *Mol Plant.* 2020; **13:** 1194-1202.
29. Benjamini Y, Hochberg Y. Controlling the false discovery rate: a practical and powerful

approach to multiple testing. *J. R. Stat. Soc. Series B Stat. Methodol.* 1995; **57:** 289-300.
30. Kleinman-Ruiz D, Lucena-Perez M, Villanueva B et al. Purging of deleterious burden in the endangered Iberian lynx. *Proc Natl Acad Sci USA* 2022; **119:** e2110614119.
31. García-Dorado A. Shortcut predictions for fitness properties at the mutation–selection–drift balance and for its buildup after size reduction under different management strategies. *Genetics.* 2007; **176:** 983-997.
32. García-Dorado A. Understanding and predicting the fitness decline of shrunk populations: inbreeding, purging, mutation, and standard selection. *Genetics.* 2012; **190:** 1461-1476.
33. Kimura M. On the probability of fixation of mutant genes in a population. *Genetics* 1962; **47:** 713.
